# GPCR-mediated glucose sensing system regulates light-dependent fungal development and mycotoxin production

**DOI:** 10.1371/journal.pgen.1008419

**Published:** 2019-10-14

**Authors:** Thaila Fernanda dos Reis, Laura Mellado, Jessica M. Lohmar, Lilian Pereira Silva, Jing-Jiang Zhou, Ana M. Calvo, Gustavo H. Goldman, Neil A. Brown

**Affiliations:** 1 Faculdade de Ciências Farmacêuticas, Universidade de São Paulo, Ribeirão Preto, Brazil; 2 Biointeractions and Crop Protection, Rothamsted Research, Hertfordshire, United Kingdom; 3 Key Laboratory of Green Pesticide and Agricultural Bioengineering, Ministry of Education, Guizhou University, Huaxi District, Guiyang, China; 4 Department of Biological Sciences, Northern Illinois University, Illinois, United States of America; 5 Department of Biology & Biochemistry, University of Bath, Claverton Down, Bath, United Kingdom; University of Wisconsin Madison, UNITED STATES

## Abstract

Microorganisms sense environmental fluctuations in nutrients and light, coordinating their growth and development accordingly. Despite their critical roles in fungi, only a few G-protein coupled receptors (GPCRs) have been characterized. The *Aspergillus nidulans* genome encodes 86 putative GPCRs. Here, we characterise a carbon starvation-induced GPCR-mediated glucose sensing mechanism in *A*. *nidulans*. This includes two class V (*gprH* and *gprI*) and one class VII (*gprM*) GPCRs, which in response to glucose promote cAMP signalling, germination and hyphal growth, while negatively regulating sexual development in a light-dependent manner. We demonstrate that GprH regulates sexual development via influencing VeA activity, a key light-dependent regulator of fungal morphogenesis and secondary metabolism. We show that GprH and GprM are light-independent negative regulators of sterigmatocystin biosynthesis. Additionally, we reveal the epistatic interactions between the three GPCRs in regulating sexual development and sterigmatocystin production. In conclusion, GprH, GprM and GprI constitute a novel carbon starvation-induced glucose sensing mechanism that functions upstream of cAMP-PKA signalling to regulate fungal development and mycotoxin production.

## Author summary

Sensing and responding to the environment is essential for the survival of all fungi. Despite our detailed knowledge of the signalling events that coordinate these adaptations, the extracellular receptors and stimuli that trigger these adaptations remain elusive. G-protein coupled receptors (GPCRs) are the largest class of receptors in fungi and regulate multiple aspects of their biology. Here, we characterized a novel GPCR-mediated glucose sensing mechanism in *Aspergillus nidulans*, which influences fungal development and toxin production. This work suggests fungal GPCRs sense glucose and propagate their signals through the cAMP-PKA and light-responsive VeA pathways to promote vegetative growth, while repressing sexual development and toxin production. Then we show how light also impacts on these nutrient sensing mechanisms. Our work reflects the complex and integrated structure of fungal mechanism to perceive different environmental stimuli, and how these mechanisms provide developmental and metabolic versatility to enable fungi to adapt to a fluctuating environment. Fungal GPCRs are poorly conserved in animals and plants, making them relevant antifungal drug targets to intervene in fungal disease and toxin contamination.

## Introduction

Sensing and responding to fluctuations in the surrounding environment, such as nutrients and light, is crucial to a microorganism's survival. In eukaryotes, G-protein signalling mechanisms play a vital role in environment sensing, and are typically composed of G-protein coupled receptors (GPCRs), a heterotrimeric G-protein complex consisting of α, β and γ subunits, and a variety of effector proteins [1, 2]. GPCRs communicate changes in the extracellular environment to intracellular G-proteins that initiate and direct signalling events to coordinate the appropriate transcriptional response. In fungi, G-protein signalling regulates cell growth and division, mating, cell-to-cell fusion, morphogenesis, chemotaxis, secondary metabolite production and virulence [3–7]. However, the functions of fungal GPCRs are less well understood and only a few putative receptor-activating ligands have been identified.

*Aspergillus nidulans* has been used in research as a model filamentous ascomycete fungus for more than six decades [8]. It is a saprophytic food spoilage mould that is phylogenetically related to *Aspergillus fumigatus*, the opportunistic pathogen of immunocompromised individuals [9]. The *A*. *nidulans* genome is predicted to encode 86 putative GPCRs, which are classified according to their structural similarities and putative activating ligands [10, 11]. Sixteen receptors, named GprA-GprP and NopA, constitute nine categories of GPCRs. Finally, 70 *A*. *nidulans* class X Pth11-like receptors, which promote fungal-plant pathogenic interactions, were identified [10, 12, 13].

Nutritional state and the perception of a sexual partner regulate sexual development. In *A*. *nidulans*, the formation of sexual structures is promoted by favourable nutrient-rich conditions and inhibited by carbon or amino acid starvation [4, 14]. The GprA and GprB pheromone receptors are required for sexual development, but not vegetative growth [15], while the putative GprD carbon receptor promotes hyphal growth and conidial germination when grown in the presence of glucose [4]. GprD-mediated carbon sensing also acts as a repressor of sexual development, and potentially functions upstream of sex pheromone signalling. But this phenotype is abolished during carbon starvation, suggesting the presence of other repressors of sex under this condition. The putative GprH carbon and amino acid receptor functions upstream of the cAMP-activated Protein Kinase A (PKA) pathway, promoting glucose uptake and hyphal growth, while repressing sexual development, during carbon starvation [16]. Therefore, the GprD and GprH mechanisms appear to operate under distinct environmental conditions and repressing sexual development.

Light also influences fungal developmental decisions [17–19], with *A*. *nidulans* predominantly producing asexual conidia in the light and sexual fruiting bodies (cleistothecia) in the dark [20–22]. The Velvet family proteins, VelB, VelC and VosA transcription factors, plus the VeA global regulator gene, are light-dependent regulators, which interact with the LaeA methyltransferase, to regulate fungal development and secondary metabolism [23]. The interaction between VeA and VelB is essential for cleistothecia formation and is established in the cytoplasm. In the dark, the dimer is transported to the nucleus [17], where VeA also interacts with LaeA, affecting sexual development [24]. Additionally, in the dark VelB binds VosA and represses asexual conidiation, while under light, LaeA reduces VosA and VelB levels, triggering asexual sporulation [25].

Development and secondary metabolism are genetically linked in *A*. *nidulans* [26, 27]. *Aspergillus nidulans* produces a variety of secondary metabolites which are toxic to humans and animals, including, sterigmatocystin (ST) [28], the penultimate precursor of aflatoxins produced by related *Aspergillus* species [29, 30]. The ST biosynthetic gene cluster is regulated by the cluster-specific AflR transcription factor [31], which operates downstream of glucose sensing, G-protein signalling and the cAMP-PKA [32]. Additionally, LaeA also regulates the ST biosynthetic gene cluster in an AflR-dependent manner. The Δ*laeA* mutation abolishes *aflR* expression and subsequently the production of ST and other secondary metabolites [33]. The cross-talk between light and glucose sensing in the coordination of ST production and fungal development is mediated by VeA [34]. Similarly, the Δ*veA* and Δ*velB* mutations that disrupt the formation of Velvet complexes impair ST production [24, 35]. GPCRs play a critical role perceiving these environmental stimuli and regulating the appropriate signalling pathways [36]. However, the importance of GPCRs and the functional connections between them in regulating secondary metabolism are unknown.

Here, we characterise a novel carbon starvation-induced GPCR mechanism in *A*. *nidulans*. This consists of three GPCRs, two class V receptors (*gprH* and *gprI*) and a single class VII (*gprM*) receptor. These GPCRs respond to glucose, promoting cAMP signalling, germination and hyphal growth, while negatively regulating sexual development in a light-dependent manner. We then show that GprH coordinates sexual development via regulating VeA nuclear localisation and activity, a key light-dependent regulator of fungal morphogenesis and secondary metabolism. Additionally, our studies revealed that GprH and M are light-independent negative regulators of sterigmatocystin biosynthesis. Genetic studies showed the epistatic interactions between the three GPCRs in the regulation of sexual development and sterigmatocystin mycotoxin biosynthesis. These studies reflect the complexity and integrated structure of fungal GPCR-mediated mechanisms in coordinating the perception of environmental stimuli with fungal development and secondary metabolism, to achieve optimal adaptations to a changing environment.

## Results

### The transcriptional regulation of GPCR encoding genes during carbon starvation

The expression of the 16 class I-IX GPCR encoding genes, named *gprA-gprP* and *nopA*, was assessed during growth on glucose and carbon starvation (Table 1). This included two class V receptors, *gprH* and *gprI*, with similarity to the *Dictyostelium discoideum* cAMP receptor cAR1 [37]. The previous genome-wide microarray study of the transcriptional response of *A*. *nidulans* to carbon starvation showed that class V receptor *gprH* was transcriptionally induced during carbon starvation, where it regulated glucose uptake, hyphal growth and repressed sexual development [16, 38]. The gene model for the other class V receptor, *gprI* (AN8348), was found to be inaccurate on chromosome V of the FGSC_A4 genome annotation within FungiDB database [39] and was not predicted to yield a 7-TM containing GPCR. The BLAT alignment of this gene model was also not in agreement with other *Aspergillus* species and closely related fungi. Using RNA-sequencing data, a modified *A*. *nidulans* AN8348_M model was corrected to encode a class V receptor with 7-TM and the Dicty_CAR domains (S1 Fig).

**Table 1:** The log2 fold change in the expression of GPCRs in *A*. *nidulans* post transfer from growth in glucose to carbon starvation for 12 and 24 h (38). N/A = No significant modulation in gene expression (*p>*0.01).

Gene name	Gene ID	Class	Domains (No. TMs)	Carbon starvtion				
				Expression	12 h		24 h	
					Mean	Std	Mean	Std
*gprA (preB)*	AN2520	I	Ste2; alpha-pheromone receptor (7-TM)	Up	1.30	0.21	1.20	0.10
*gprB (preA)*	AN7743	II	Ste3; a-pheromone receptor (5-TM)	N/A				
*gprC*	AN3765	III	Git3; Gpa2 C-term; Family 1-like (7-TM)	N/A				
*gprD*	AN3387	III	Git3; Gpa2 C-term (7-TM)	N/A				
*gprE*	AN9199	III	Git3; Gpa2 C-term (7-TM)	Up	0.52	0.10	0.53	0.08
*gprF*	AN12206	IV	PQ-loop repeat (7-TM)	Down	-1.88	0.20	-2.08	0.11
*gprG*	AN10166	IV	PQ-loop repeat (5-TM)	Up	0.81	0.11	0.81	0.08
*gprH*	AN8262	V	Secretin-like; Dicty_CAR; Family 2-like (7-TM)	Up	1.83	0.21	1.86	0.21
*gprI* *	AN8348	V	Dicty_CAR; Family 2-like (7-TM)	Absent				
*gprJ*	AN5720	IV	PQ-loop repeat (7-TM)	Down	-1.43	0.02	-1.26	0.03
*gprK*	AN7795	VI	GprK-like; RGS (7-TM)	Down	-1.34	0.09	-1.81	0.16
*gprM*	AN6680	VII	MG00532-like (7-TM)	Up	0.49	0.07	0.72	0.05
*gprN*	AN5508	VII	MG00532-like (6-TM)	N/A				
*gprO*	AN4932	VIII	mPR-like; Haemolysin-III related (7-TM)	Up	0.81	0.21	0.97	0.38
*gprP*	AN5151	VIII	mPR-like; Haemolysin-III related (7-TM)	N/A				
*nopA*	AN3361	IX	Bacterial rhodopsin-like (6-TM)	Down	-3.44	0.14	-2.73	0.22

* GprI gene model corrected to ChrV, antisense, 194574–1957377. Therefore, expression data is absent from array.

Among the remaining GPCR encoding genes, the *gprA* α-pheromone receptor was induced despite carbon starvation representing a condition repressive of sexual development. The three putative class III carbon receptors *gprC-E* did not show a major increase in expression. Among the three class IV putative nitrogen receptors possessing a PQ-loop *gprF* and *gprJ* were highly repressed, while *gprG* which only is predicted to encode 5-TMs was induced. The class VI *gprK* receptor with the cytoplasmic repressor G-protein signalling domain, and the class IX opsin receptor, were repressed during carbon starvation. Single class VII (*gprM*) and VIII (*gprO*) receptors were induced, however, the latter showed greater variation in expression. Therefore, *gprI* which represented another uncharacterized class V receptor and *gprM* which represented an uncharacterized receptor consistently induced by carbon starvation, were selected to future investigation.

**Fig 1:**
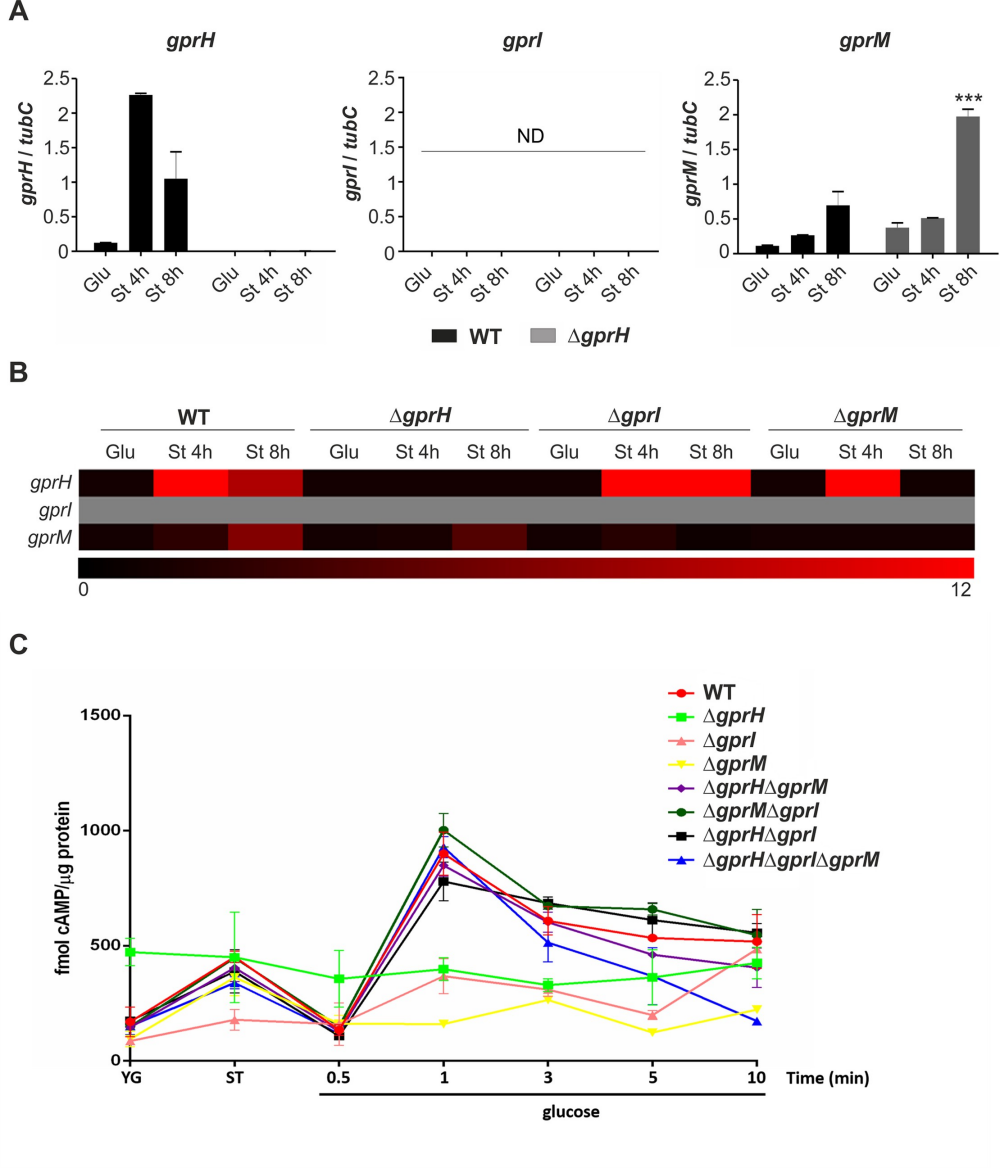
Carbon starvation induced *gprH*, *gprI* and *gprM* expression, which influence cAMP signalling. **A-B)** Conidia of the wild-type (WT), Δ*gprH*, Δ*gprI* and Δ*gprM* strains were grown in MM with 1% glucose, then subsequently washed and transferred to MM lacking a carbon source for 4 and 8 h (short-term carbon starvation). Steady-state mRNA levels of each individual *gpr* (*gprH*, *gprI* and *gprM)* were measured by RT-qPCR and normalized with *tubC*. **A)** Expression levels of *gprH*, *gprI* and *gprM* in the WT and Δ*gprH* strains are presented as copy number ratio relative to the reference gene. Statistical analysis was performed using two-way ANOVA (Analysis of Variance) with Bonferroni post-test when compared to the WT strain in each condition (* *p*< 0.05). **B)** Heat map representing the mRNA accumulation of the *gpr* genes during carbon starvation, corresponding to the WT, Δ*gprH*, Δ*gprI* and Δ*gprM* strains. Data presented as fold induction. Grey indicates expression not detected. Glu indicates glucose, St indicates starvation. **C)** Conidia of the corresponding *gpr* null mutants and the WT strain were grown in YG for 16 h, then mycelia were washed and incubated in MM lacking a carbon source for 4 h. Glucose at final concentration of 2% was added and incubated for up to 10 min. The results are the average of two biological replicates (with two technical repetitions). Statistical analysis was carried out using two-way ANOVA test with Bonferroni post-test when compared to the WT strain (* *p*< 0.05, ** *p*< 0.01, *** *p*< 0.001). cAMP levels presented as fmol/μg protein.

We hypothesized that these three receptors (*gprH*, *gprI* and *gprM*) may represent a nutrient sensing mechanism that is activated during carbon starvation. To investigate their functions and the potential genetic interactions between these receptors, the Δ*gprH*, Δ*gprI* and Δ*gprM* null mutant strains were generated in the *A*. *nidulans* AGB551 wild-type, *veA+*, background (S1 Table). The expression of the three GPCR encoding genes during carbon starvation in the wild-type strain, and its absence in the mutant strains, was confirmed via RT-qPCR. In the wild-type strain, *gprH* and *gprM* were transcriptionally induced after the shift to carbon starvation, where *gprH* showed the highest level and fastest rate of induction, while *gprI* expression was not detected (Fig 1A), validating the previously observed microarray data.

The dependency of the transcriptional induction of individual GPCRs during carbon starvation on the presence of the other two GPCRs was assessed (Fig 1B). The absence of *gprI* or *gprM* lead to the increased expression of the *gprH* during carbon starvation. Similarly, *gprM* showed increased expression in the Δ*gprH* mutant after 8 h of carbon starvation. However, no *gprI* expression was detected in any of the strains. The increased expression of different GPCRs in the absence of others, implies that functional redundancy and compensatory functions may exist among these three GPCRs during carbon starvation.

### Identification of GprH, GprI and GprM as a carbon starvation-induced, putative carbon sensing mechanism regulating cAMP signalling

We previously showed that the absence of GprH affected the production of a glucose-induced cAMP burst, glucose uptake and the recovery of growth in starved germlings, suggesting that GprH sensed glucose and functioned upstream of the cAMP-PKA pathway [16]. Accordingly, we quantified intracellular cAMP levels in the Δ*gprH*, Δ*gprI* and Δ*gprM* strains during carbon starvation and after the re-introduction of glucose. In the wild-type strain, the addition of glucose resulted in a burst in cAMP, a response that was absent in both the Δ*gprI* and Δ*gprM* strains (Fig 1C). This was reminiscent of the absence of a glucose-induced cAMP burst in the Δ*gprH* strain. This suggests that GprH, GprI and GprM are all putative carbon sensors functioning upstream of the cAMP-PKA pathway in *A*. *nidulans*. Double (Δ*gprH*Δ*gprI*, Δ*gprH*Δ*gprM*, Δ*gprI*Δ*gprM*) and triple (Δ*gprH*Δ*gprI*Δ*gprM*) mutants were used to investigate the possible genetic interaction between the three receptors in the regulation of cAMP signalling post carbon starvation. The simultaneous absence of multiple GPCRs, in the double or triple null mutants, resulted in a recovery of the burst in cAMP production post the addition of glucose (Fig 1C). This suggests that the absence of multiple GPCRs may activate a compensatory cAMP signalling mechanism.

### GprH, GprI and GprM influence conidial germination and hyphal growth in response to glucose

**Fig 2:**
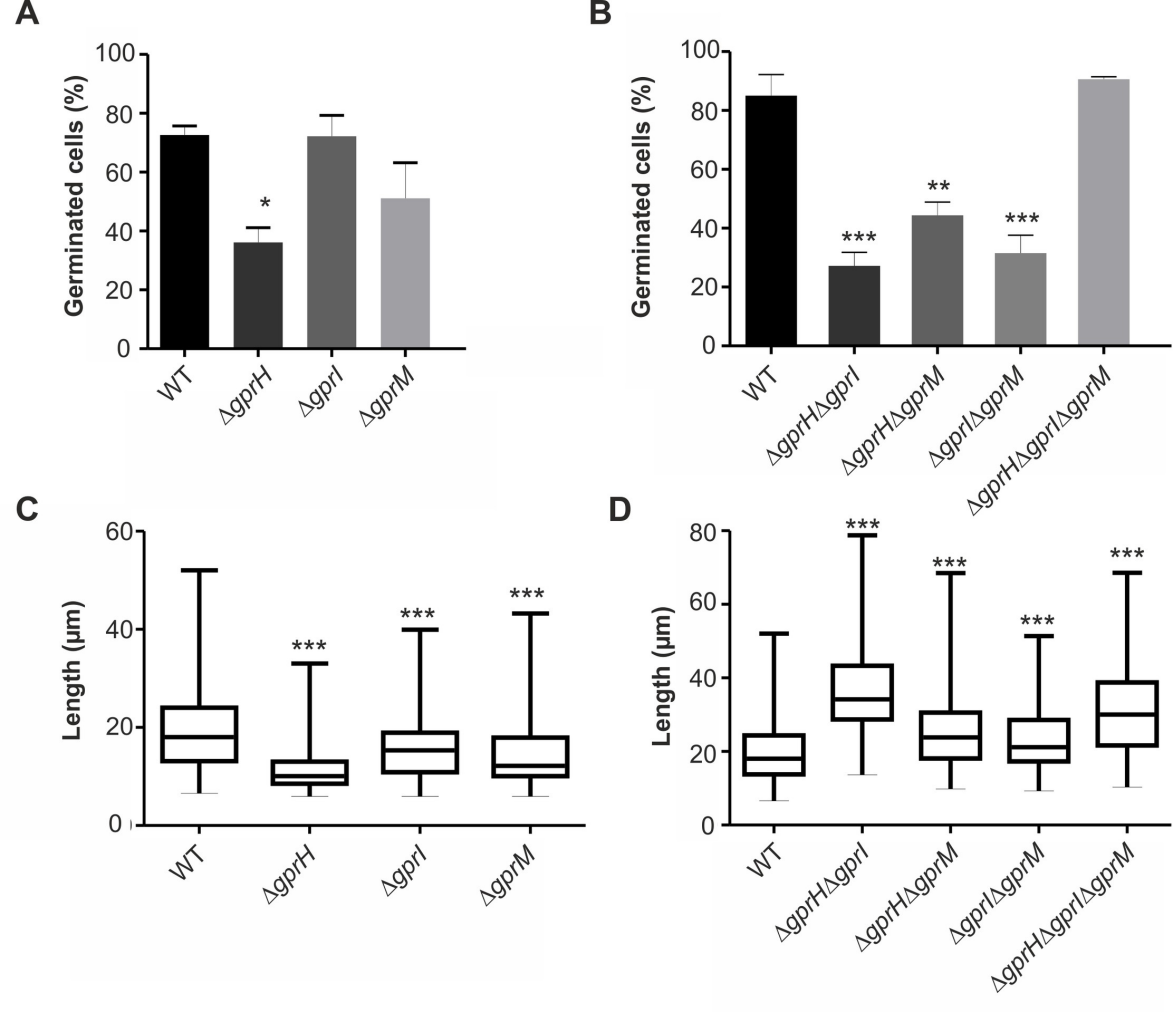
GprH, GprI and GprM influence conidial germination and hyphal growth in response to glucose. **A-B)** Conidia of WT and *gpr* null mutants were incubated in MM with glucose 1% for 6 h at 37°C. A total of 100 germlings were counted (three biological independent experiments). Statistical analysis was performed using one-way ANOVA with Tukey's post hoc test compared to the WT strain * *p*< 0.05, ** *p*< 0.01, *** *p*< 0.001). **A)** Percentage of germinated cells corresponding to WT, Δ*gprH*, Δ*gprI* and Δg*prM* strains. **B)** Percentage of germinated cells corresponding to WT, Δ*gprH*Δ*gprI*, Δ*gprH*Δ*gprM*, Δ*gprI*Δ*gprM* and Δ*gprH*Δ*gprI*Δ*gprM* strains. **C-D)** The *A*. *nidulans* WT and *gpr* null mutant strains were cultured in MM with 2% glucose for 3 h, then washed and shifted into media containing no glucose for 3 h. Finally, they were transferred to media containing 2% glucose for 1 h. The length (μm) of 300 germlings (100 cells in 3 independent biological repetitions) was measured. Statistical analysis was performed using Mann–Whitney test (*** *p<* 0.001). **C)** Length of germlings corresponding to WT, Δ*gprH*, Δ*gprI* and Δ*gprM* strains after the re-introduction of glucose. **D)** Length of germlings corresponding to WT, Δ*gprH*Δ*gprI*, Δ*gprH*Δ*gprM*, Δ*gprI*Δ*gprM* and Δ*gprH*Δ*gprI*Δ*gprM* strains after the re-introduction of glucose.

In *A*. *nidulans* the cAMP-PKA pathway positively regulates conidial germination in the presence of glucose [40]. Subsequently, we determined if the three putative glucose receptors also influenced conidial germination in the presence of glucose. The Δ*gprH* strain showed a reduction in germination frequency in comparison to the wild-type strain, suggesting that *gprH* may positively regulate conidial germination (Fig 2A). No differences in germination frequency were observed for the Δ*gprI* or Δ*gprM* strains. The combination of Δ*gprH* with either Δ*gprI* or Δ*gprM*, decreased germination frequency compared to the wild-type strain, to a similar extent as the single Δ*gprH* strain (Fig 2B). Interestingly, combining the Δ*gprI* and Δ*gprM* mutations also decreased conidial germination, a phenotype that was absent in the corresponding single null mutants, suggesting the occurrence of an additive genetic effect and redundancy between the two receptors. However, the combination of all three Δ*gprH*, Δ*gprI* and Δ*gprM* deletions restored germination frequency to wild-type levels. Collectively, this suggests that the GprH signalling mechanism may function in parallel to GprI and GprM, which function in epistasis, to regulate conidial germination in response to glucose, while the absence of all three receptors may activate a compensatory mechanism.

The cAMP-PKA pathway in *A*. *nidulans* also positively regulates vegetative hyphal growth [6, 40]. GprH was previously shown to influence glucose uptake and the recovery of growth in starved germlings [16]. The recovery of hyphal growth after starvation and the re-introduction of glucose was assessed to determine if GprH, GprI and GprM genetically interact during these conditions. As we previously described for the Δ*gprH* strain, the Δ*gprI* and Δ*gprM* strains also displayed a delay in the recovery of growth after the re-introduction of glucose to starved cells (Fig 2C). Analysis of the double and the triple null GPCR mutants unexpectedly revealed that the combination of two, or the three, null GPCR mutations did not impair hyphal growth, and in fact showed increased germling length after the re-introduction of glucose (Fig 2D). This reflects the recovery of glucose-induced cAMP signalling in the simultaneous absence of multiple GPCRs. Taken together, these data suggest that GprH, GprI and GprM represent carbon starvation-induced glucose sensing mechanisms that promote germination and hyphal growth in *A*. *nidulans*. However, the absence of multiple receptors activates an unknown compensatory mechanism that promotes cAMP signalling and hyphal growth post starvation.

### GprH, GprI and GprM influence light-dependent regulation of asexual and sexual development

**Fig 3:**
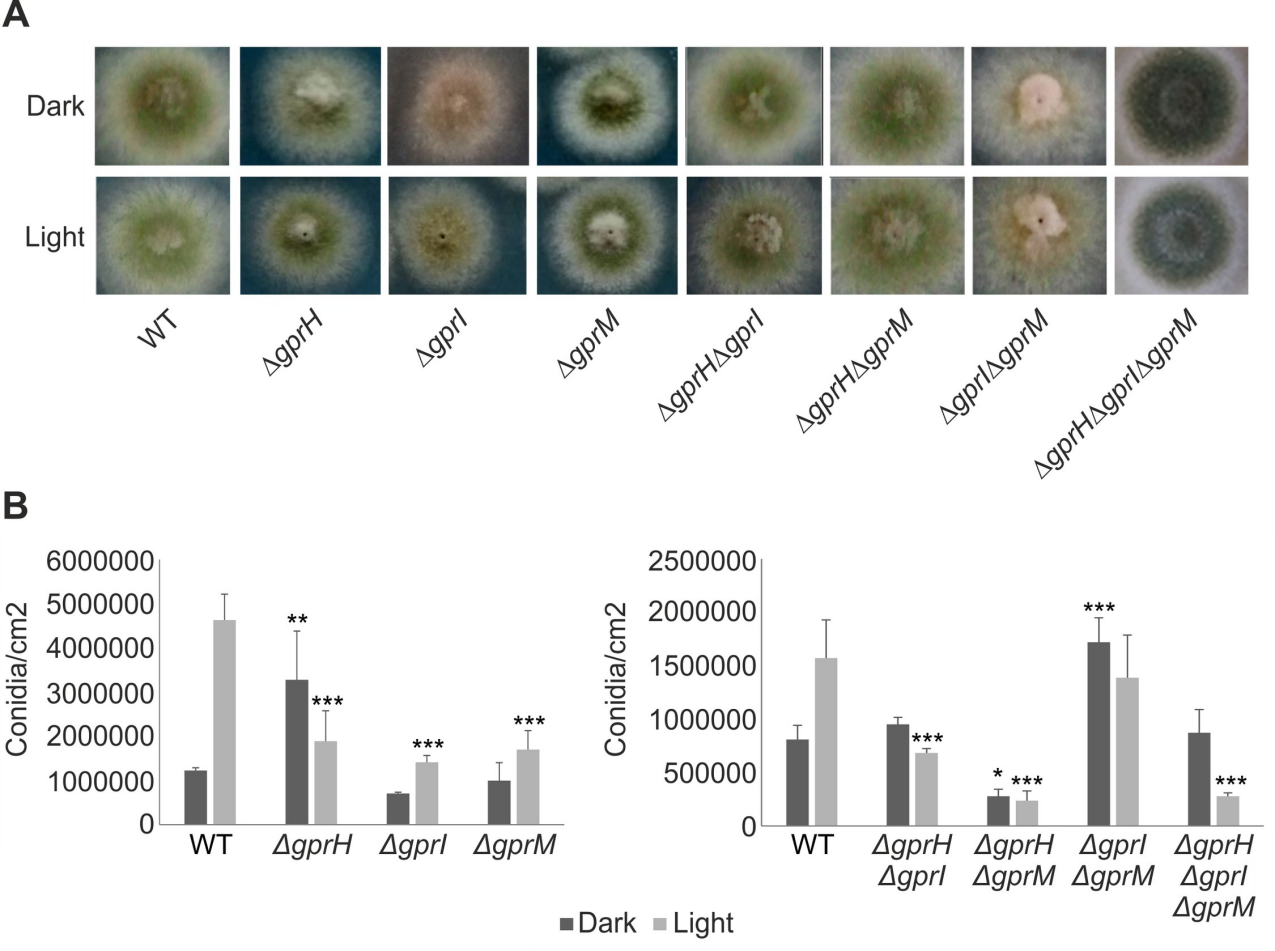
The influence of GprH, GprI and GprM on vegetative growth and asexual conidiation in the presence of light or darkness. The *A*. *nidulans* wild-type (WT), Δ*gprH*, Δ*gprI*, Δ*gprM*, Δ*gprH*Δ*gprI*, Δ*gprH*Δ*gprM*, Δ*gprI*Δ*gprM* and Δ*gprH*Δ*gprI*Δ*gprM* strains were grown in the presence of light and dark to assess radial growth and asexual conidiation. Conidia were grown on solid MM containing glucose, incubated at 37°C for 24 h in darkness and transferred to either constant white light or darkness for 48 h. **A)** No mutants showed significant alteration to radial growth. **B)** The number of conidia/cm^2^ determined using a Neubauer chamber. Statistical analysis was performed using a two-way ANOVA with Bonferroni post-test when compared to the wild-type strain in each condition (*** *p*< 0.001, ** *p*< 0.01, * *p*< 0.05).

The presence of light influences fungal development, where *A*. *nidulans* favours asexual conidiation in the light and sexual development in the dark [22]. The *A*. *nidulans* wild-type and mutant strains were grown in the presence of light and dark to assess radial growth and asexual conidiation. None of the mutants showed a significant alteration in radial growth (Fig 3A). The wild-type strain produced significantly more asexual conidia in the presence of light than in the dark (Fig 3B). The opposite was true for the Δ*gprH* mutant, which produced more conidia in the dark and showed reduced conidia production in the light. The Δ*gprI* and Δ*gprM* mutants both produced more conidia in the light than dark, but fewer than the wild-type strain. The combination of Δ*gprH* with Δ*gprI*, Δ*gprM*, or both Δ*gprI*Δ*gprM*, reduced conidiation in the light. However, the combination of Δ*gprI* and Δ*gprM* did not reduced conidiation in the light, but instead increased conidiation in the dark.

**Fig 4:**
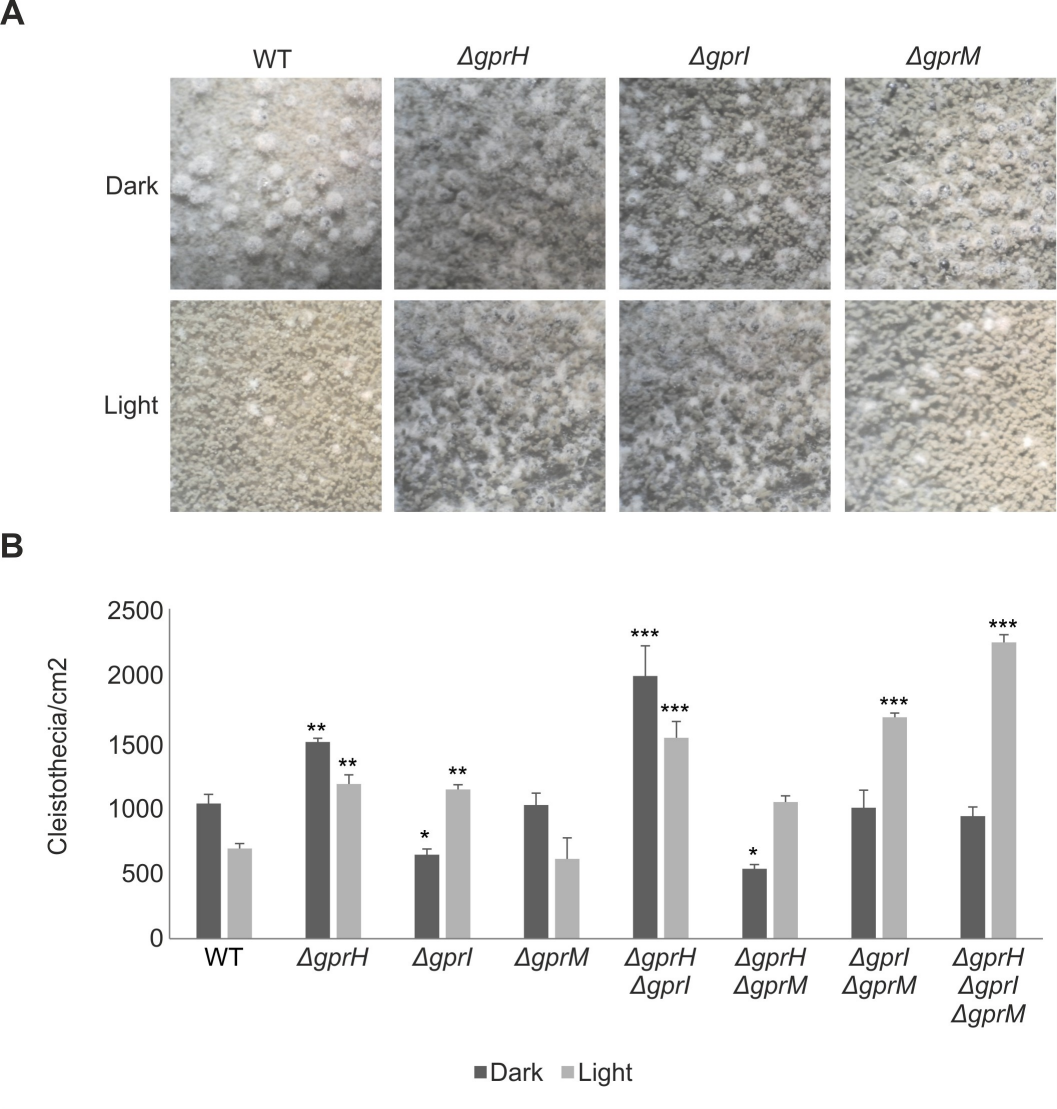
GprH, GprI and GprM regulate sexual development on glucose in a light-dependent manner. Conidia of the *A*. *nidulans* wild-type (WT), Δ*gprH*, Δ*gprI*, Δ*gprM*, Δ*gprH*Δ*gprI*, Δ*gprH*Δ*gprM*, Δ*gprI*Δ*gprM* and Δ*gprH*Δ*gprI*Δ*gprM* strains were grown on solid MM and incubated at 37°C for 24 h in darkness. **A)** Plates were sealed and transferred into either constant white light or darkness for 96 h to enable cleistothecia (black spherical sexual fruiting bodies) to develop. **B)** The number of cleistothecia/cm^2^ was determined. Statistical analysis was performed using a two-way ANOVA with Bonferroni post-test when compared to the wild-type strain in each condition (*** *p*< 0.001, ** *p*< 0.01, * *p*< 0.05).

The production of sexual fruiting bodies, termed cleistothecia, was assessed on sealed minimal media plates incubated in the light or dark (Fig 4A). Under dark conditions inductive of sexual development, Δ*gprH* increased cleistothecia production (Fig 4B). In the presence of light, Δ*gprH* and Δ*gprI* increased cleistothecia production. The combination Δ*gprH*Δ*gprI* had an additive effect and increased cleistothecia production in the light or dark. However, Δ*gprM* did not affect sexual development in the light or dark, and when combined with Δ*gprH* wild-type cleistothecia production was restored. But cleistothecia production remained elevated in the light when the Δ*gprM* and Δ*gprI* mutations were combined. Cleistothecia production was maximal in the triple Δ*gprH*Δ*gprI*Δ*gprM* mutant in the presence of light. Collectively, this again suggests that the GprH signalling mechanism functions in parallel to GprI and GprM, which function in epistasis, to regulate fungal developmental decisions in a light-dependent manner.

### GprH, GprI and GprM mediated, light-dependent, regulation of sexual development during carbon starvation

**Fig 5:**
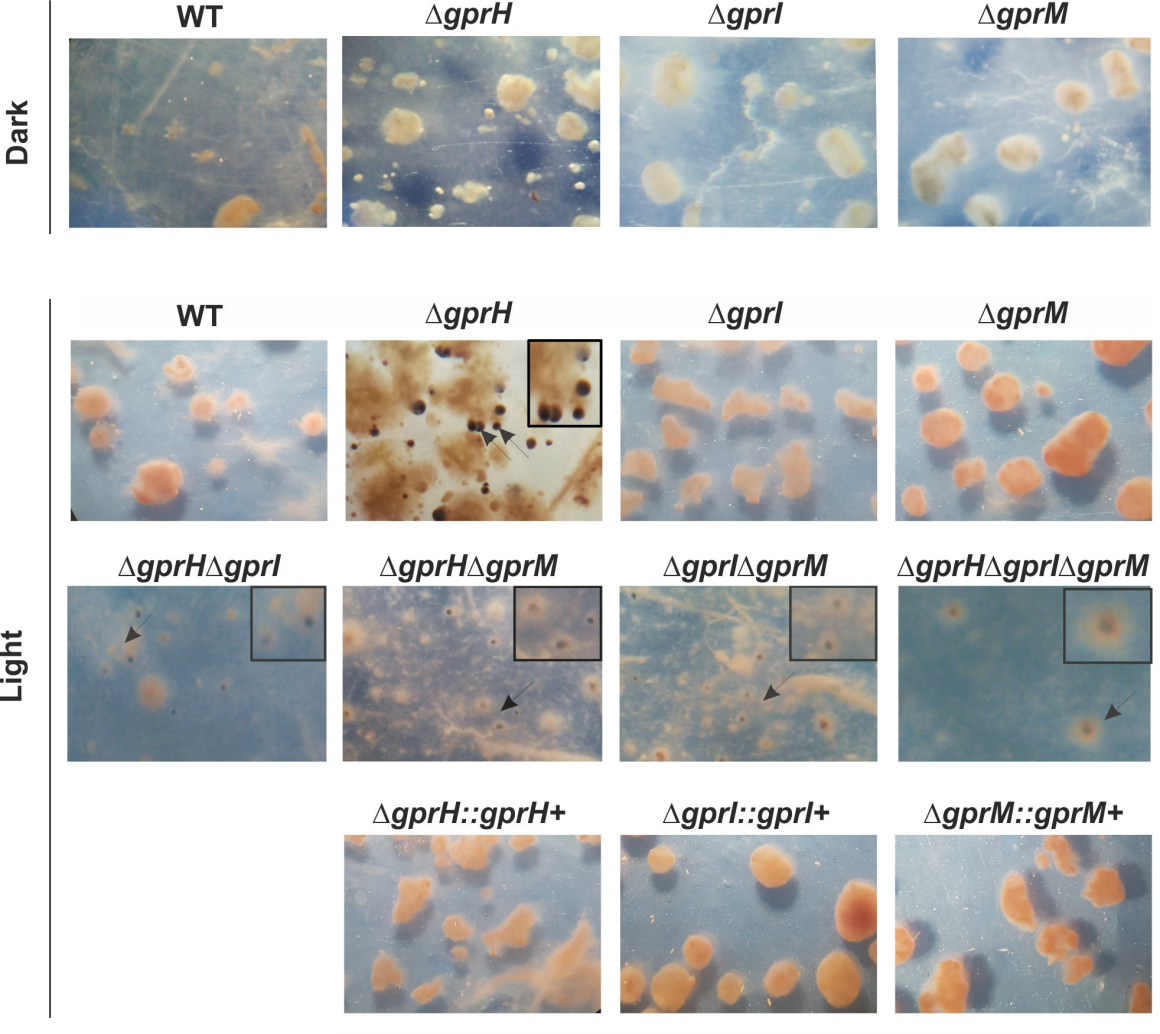
GprH, GprI and GprM mediated, light-dependent, regulation of sexual development during carbon starvation. The *A*. *nidulans* wild-type (WT), Δ*gprH*, Δ*gprI*, Δ*gprM*, Δ*gprH*Δ*gprI*, Δ*gprH*Δ*gprM*, Δ*gprI*Δ*gprM* and Δ*gprH*Δ*gprI*Δ*gprM* strains were grown in MM with glucose 2% (w/v) at 37°C for 24 h. Washed mycelia were transferred to MM containing no carbon source for 8 days, exposed either to white light or darkness. Cleistothecia formation was observed only in submerged cultures exposed to light after 8 days post-carbon starvation, in the Δ*gprH*, Δ*gprH*Δ*gprI*, Δ*gprH*Δ*gprM*, Δ*gprI*Δ*gprM* and Δ*gprH*Δ*gprI*Δ*gprM* strains. The repression of cleistothecia production during carbon starvation in the presence of light was restored by complementation of the *gprH+* allele. Arrows = clesitothecia magnified in box on top right-hand side.

Nutrient availability also determines if *A*. *nidulans* undergoes either sexual or asexual reproduction [41]. High glucose concentrations promote the production of higher numbers of sexual cleistothecia [41, 42], while light inhibits sexual reproduction [43, 44]. Accordingly, GprH was demonstrated to be a repressor of sexual development during carbon starvation (16), while this starvation condition rescued the developmental defects of the Δ*gprD* mutant [4]. The atypical production of cleistothecia in carbon starved Δ*gprH* cultures was found to be dependent on the presence of light. Subsequently, we assessed cleistothecia production in carbon-starved submerged cultures, exposed to light or dark conditions, for the Δ*gprH*, Δ*gprI* and Δ*gprM* single, double and triple null mutants (Fig 5). Similar to the wild-type strain, neither the Δ*gprI* nor Δ*gprM* strains produced cleistothecia under light or dark carbon-starved submerged conditions. Complementation of the single Δ*gprH*, Δ*gprI* and Δ*gprM* mutants with the native gene restored wild-type regulation of sexual development in carbon starved cultures (Fig 5) confirming the phenotype was dependent on the Δ*gprH* mutation. The combined absence of any two, or all three, of the GPCRs promoted cleistothecia production in carbon-starved light-exposed submerged cultures, even when combining the Δ*gprI* and Δ*gprM* mutations, despite the individual Δ*gprI* or Δ*gprM* strains failing to produce cleistothecia (Fig 5 and Table 2). This additive effect supports the conclusion that GprI and GprM may function in epistasis, and in parallel to GprH, in the regulation of sexual development during carbon starvation in the presence of light.

**Table 2:** Cleistothecia production in carbon starved submerged cultures exposed to light or dark. Visual qualitative analysis of the absence (-) or abundance (+) of cleistothecia in the culture media.

Strain	WT	*ΔgprH*	*ΔgprI*	*ΔgprM*	*ΔgprHΔgprI*	*ΔgprHΔgprM*	*ΔgprIΔgprM*	*ΔgprHΔgprlΔprM*
**Light**	-	+++	-	-	+++	+++	+++	++
**Dark**	-	-	-	-	-	-	-	-

### GprH represses sexual development by influencing VeA nuclear localization

In *A*. *nidulans* cleistothecia production predominantly occurs in the dark, while conidiophores are produced in the light [22]. The VeA Velvet protein positively regulates sexual development in a light-dependent manner [26]. In dark, the VeA-VelB complex is imported into the nucleus to activate sexual development. VelB binds to VosA to inhibit asexual development, while the VeA-VelB-LaeA complex activates secondary metabolism [25]. Under light, the transport of VeA to the nucleus is inhibited [17], and as result VelB supports asexual conidiation, while LaeA activity is decreased [24, 45].

**Fig 6:**
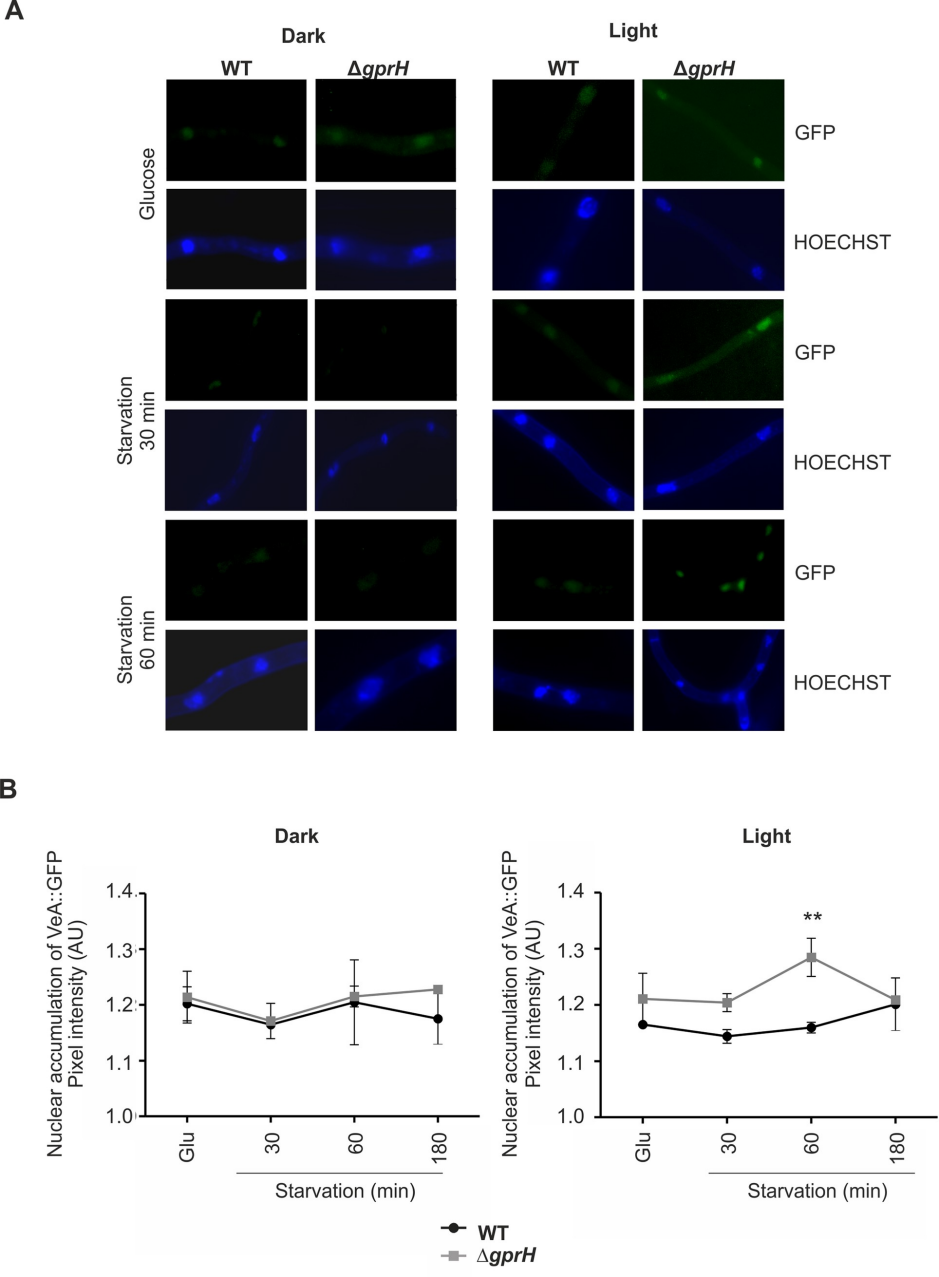
Fluorescence microscopy shows GprH regulates VeA::GFP nuclear localisation during carbon starvation in the presence of light. **A)** The subcellular localization of the VeA::GFP fusion protein. Conidia of the wild-type TRMD3.4.17 (WT) and the Δ*gprH* strains expressing *veA*::*GFP* were germinated in MM (glucose 1%), then shifted to MM with no carbon, either constantly exposed to white light or in the dark. Nuclei were stained with Hoechst to verify the VeA::GFP nuclear localization. **B)** Graphs showing the quantification of the nuclear fluorescence corresponding to VeA::GFP, in the *gprH*^*+*^ and ⨂*gprH* backgrounds. For each condition, 20 nuclei were measured. The error bars indicate standard error. Each experiment was repeated twice. Statistical analysis was performed using a two-way ANOVA with Bonferroni post-test when compared to the wild-type strain in each condition (** *p*< 0.01).

**Fig 7:**
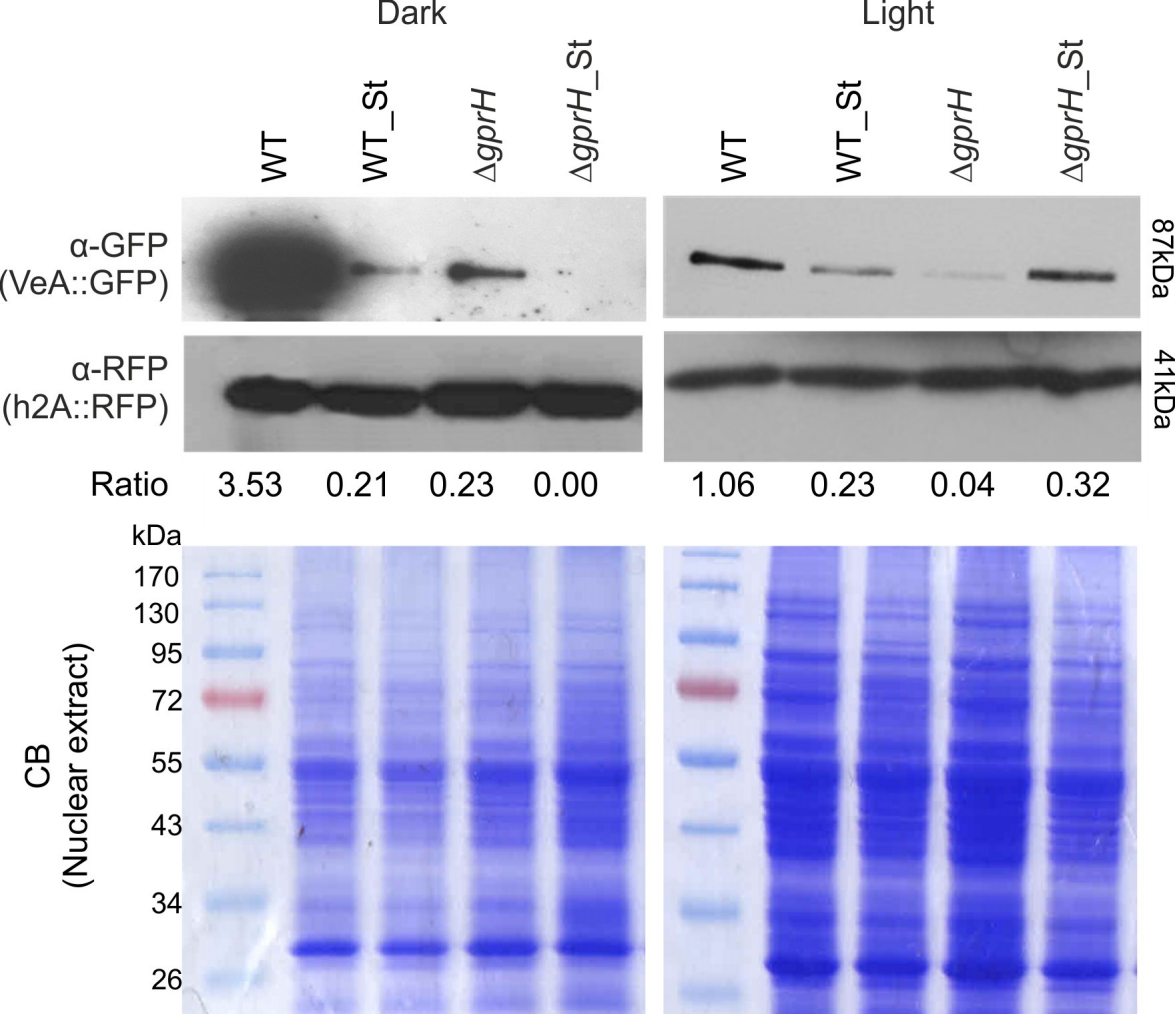
Immunoblots confirm GprH regulates VeA::GFP nuclear localisation during carbon starvation in the presence of light. The nuclear histone h2A of the wild-type (WT) and Δ*gprH* VeA::GFP strains were N-terminal tagged with mRFP (mRFP::h2A). Nuclei were isolated after growth on glucose or during carbon starvation, when exposed to light or dark conditions. Immunoblots confirmed histone isolation and the presence of VeA::GFP in the nucleus. Coomassie (CB) stained gels show consistent loading of nuclear extracted proteins.

The subcellular localization of VeA was evaluated in the wild-type TRMD3.4.17 (*gprH*^*+*^) and mutant Δ*gprH* strains, when subjected to carbon starvation under light or dark conditions. The introduction of VeA::GFP into both the TRMD3.4.17 and Δ*gprH* strains did not impact on colony growth or conidiation [17], indicating that the VeA::GFP fusion protein was functional. In the wild-type TRMD3.4.17 strain, VeA::GFP nuclear localization did not increase post carbon starvation under light or dark conditions (Fig 6A and 6B). However, in the Δ*gprH* mutant, VeA::GFP showed increased nuclear accumulation between 30 min and 1 h carbon starvation when exposed to light (Fig 6A and 6B). The nuclear histone h2A of the wild-type and Δ*gprH* VeA::GFP strains were subsequently N-terminal tagged with mRFP (mRFP::h2A). Nuclei were isolated after growth on glucose or during carbon starvation, when exposed to light or dark conditions. Immunoblots confirmed histone isolation and the presence of VeA::GFP in the nucleus. The wild-type strain revealed abundant VeA::GFP nuclear localisation when grown on glucose in the dark, and this was greatly reduced during carbon starvation (Fig 7). The Δ*gprH* mutant showed a lower overall level of VeA::GFP, but a similar regulatory pattern to the wild-type strain when exposed to starvation in the dark. However, in the presence of light, the wild-type strain showed reduced VeA::GFP levels, but an overall similar regulatory pattern of VeA::GFP localisation during carbon starvation. Conversely, the Δ*gprH* mutant showed the opposite regulatory pattern, where VeA::GFP nuclear localisation increased during carbon starvation in the light, correlating with the fluorescence imaging. Therefore, GprH does regulate VeA nuclear localisation and in turn sexual development during carbon starvation in the presence of light.

**Fig 8:**
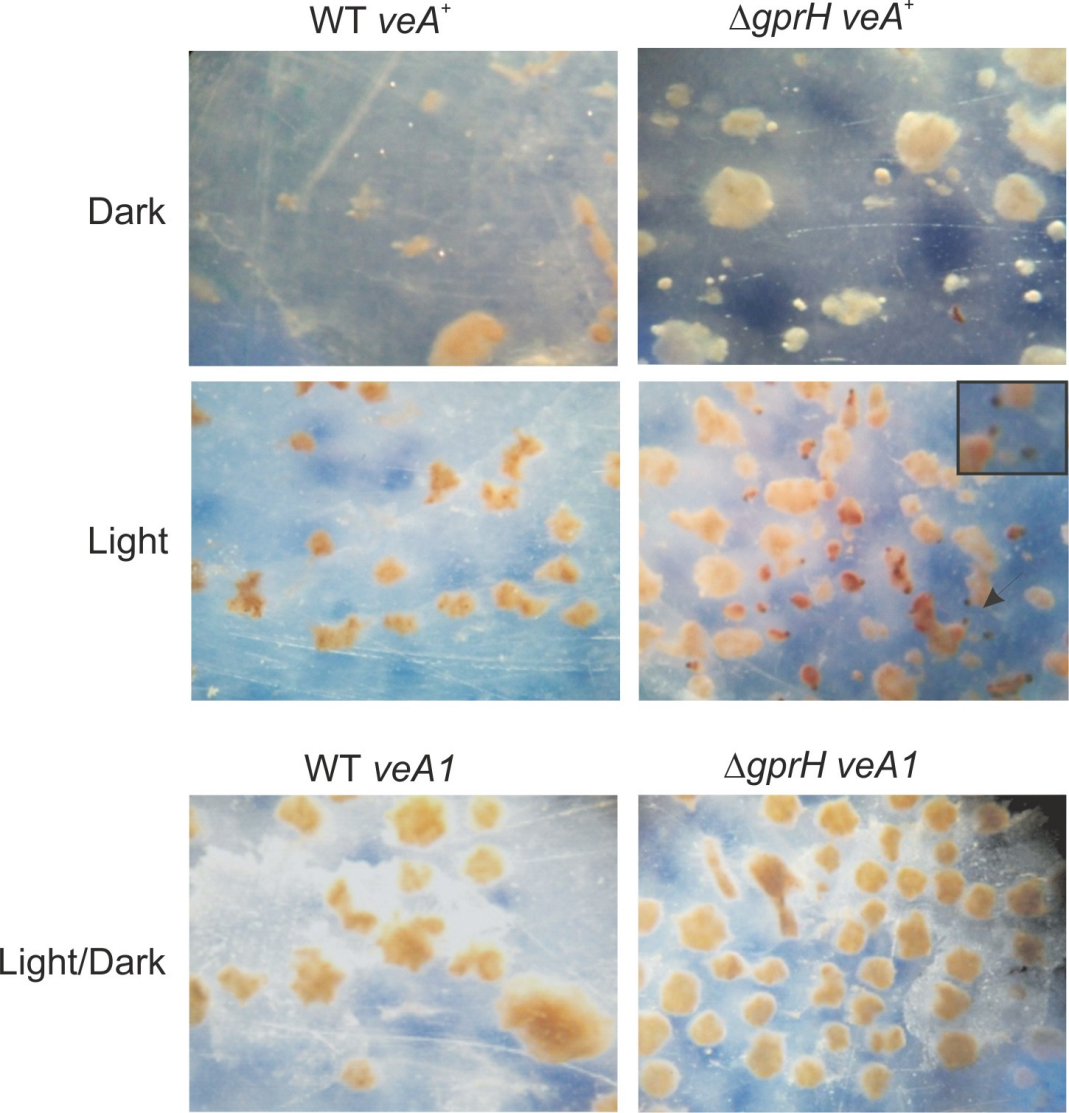
Fully functional VeA is required for hyperactivation of sexual development in Δ*gprH* strain. Cleistothecia production was only observed in carbon-starved cultures exposed to light in the Δ*gprH* mutant carrying the fully functional *veA*^+^ allele, and was absent in the Δ*gprH* mutant that possessed the partially functional *veA1* mutation. Cleistothecia were never observed in the wild-type (WT) strain under any carbon starvation conditions. This shows GprH regulates sexual development via influencing the VeA function. Arrow = clesitothecia magnified in box on top right-hand side.

The allelic *veA1* mutation of the *veA* gene, delays and reduces cleistothecia production [46]. The VeA1 protein lacks the first 36 amino acids at the N-terminus, including the nuclear localization signal, predominantly resides in the cytoplasm [17], and fails to properly regulate sexual development. To examine whether the increased production of cleistothecia observed in the Δ*gprH* mutant was dependent on VeA, a Δ*gprH* strain carrying the *veA1* mutation was generated (Fig 8). Cleistothecia production was only observed in carbon-starved cultures exposed to light in the Δ*gprH* mutant carrying the fully functional *veA*^+^ allele, and was absent in the Δ*gprH* mutant that possessed the *veA1* mutation. Collectively, these data indicate that GprH regulates sexual development via influencing the function of the VeA protein in response to light.

### GprH, GprI and GprM have differing effects on ST toxin production

In addition to regulating fungal development, the interaction between LaeA and VeA also coordinates secondary metabolism, including ST and penicillin production, with the presence of light [24, 26, 35, 47]. Among the ST cluster genes, *stcU* is one of the most highly transcribed, and the regulatory *aflR* gene activates the ST gene cluster that leads to the production of ST [31]. In our study, GprH was shown to influence VeA localization in light exposed, carbon-starved cells. Therefore, the role of GprH, as well as that of GprI and GprM, in regulating the production of ST and penicillin was assessed. In *A*. *nidulans*, the effect of light on the production of ST depends on the glucose concentration [34].

**Fig 9:**
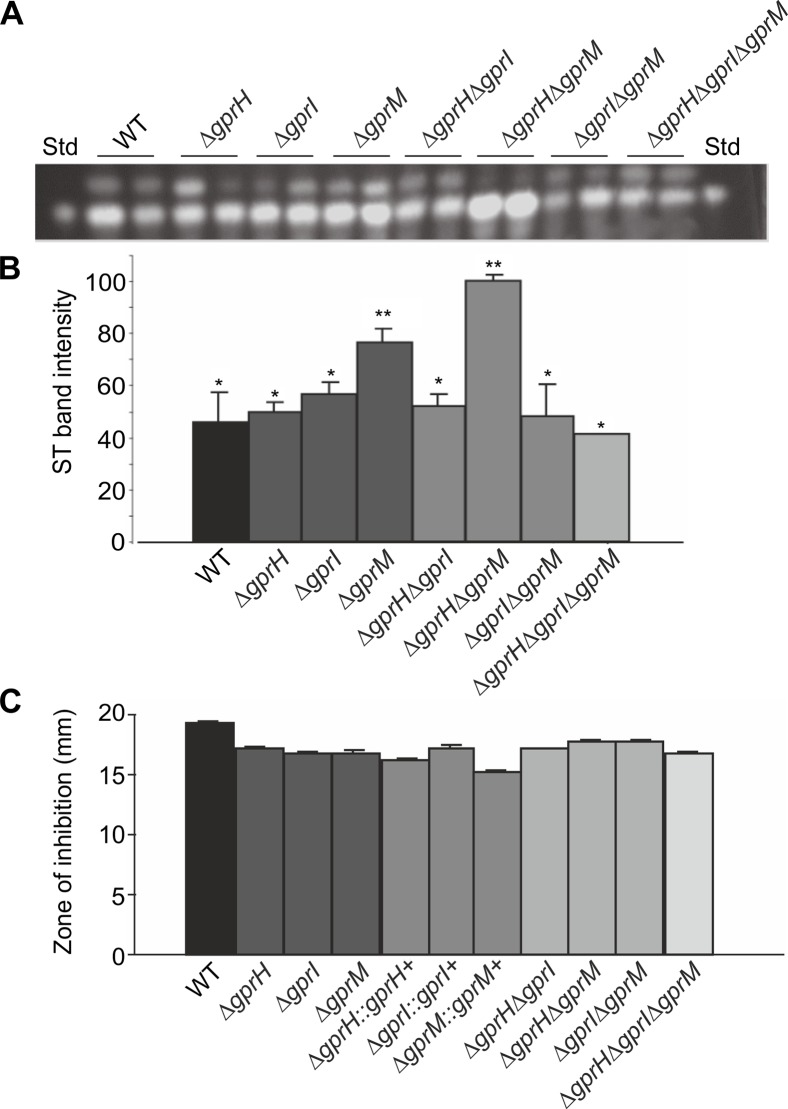
The effect of GPCRs on ST production and expression of ST regulatory genes. **A-B)** The *A*. *nidulans* wild-type (WT), Δ*gprH*, Δ*gprI*, Δ*gprM*, Δ*gprH*Δ*gprI*, Δ*gprH*Δ*gprM*, Δ*gprI*Δ*gprM* and Δ*gprH*Δ*gprI*Δ*gprM* strains were incubated in MM with glucose 1% (w/v) liquid stationary cultures at 37°C in the dark for 7 days. Presented is the TLC (A) and densitometry (B) analyses of ST production. Statistical analysis was carried out using two-way ANOVA in conjunction with Tukey's post hoc test (* *p*< 0.05). **C)** The *A*. *nidulans* wild-type (WT), Δ*gprH*, Δ*gprI*, Δ*gprM*, Δ*gprH*Δ*gprI*, Δ*gprH*Δ*gprM*, Δ*gprI*Δ*gprM* and Δ*gprH*Δ*gprI*Δ*gprM* strains were assessed for penicillin production. The antibacterial activity of the fungal supernatant was determined by measuring the zones of inhibition *B*. *calidolactis* C953. No significant difference was observed.

The epistatic interactions between GprH, GprI and GprM were examined during incubation under ST-inducing conditions, i.e. static growth on glucose in the dark for 7 days. ST production remained unchanged in the ⨂*gprH* and ⨂*gprI* mutants, but was moderately increased in ⨂*gprM* (Fig 9A and 9B). Combining the ⨂*gprM* and ⨂*gprH* mutations had an additive effect and further increased ST production. However, any combination with ⨂*gprI*, including ⨂*gprI*⨂*gprM* or ⨂*gprH*⨂*gprI*⨂*gprM*, had the opposite effect and returned ST production to wild-type levels. This suggests that GprM is a major, while GprH is a minor, repressor of ST production, and that the elevated production of ST in their absence was dependent on GprI.

**Fig 10:**
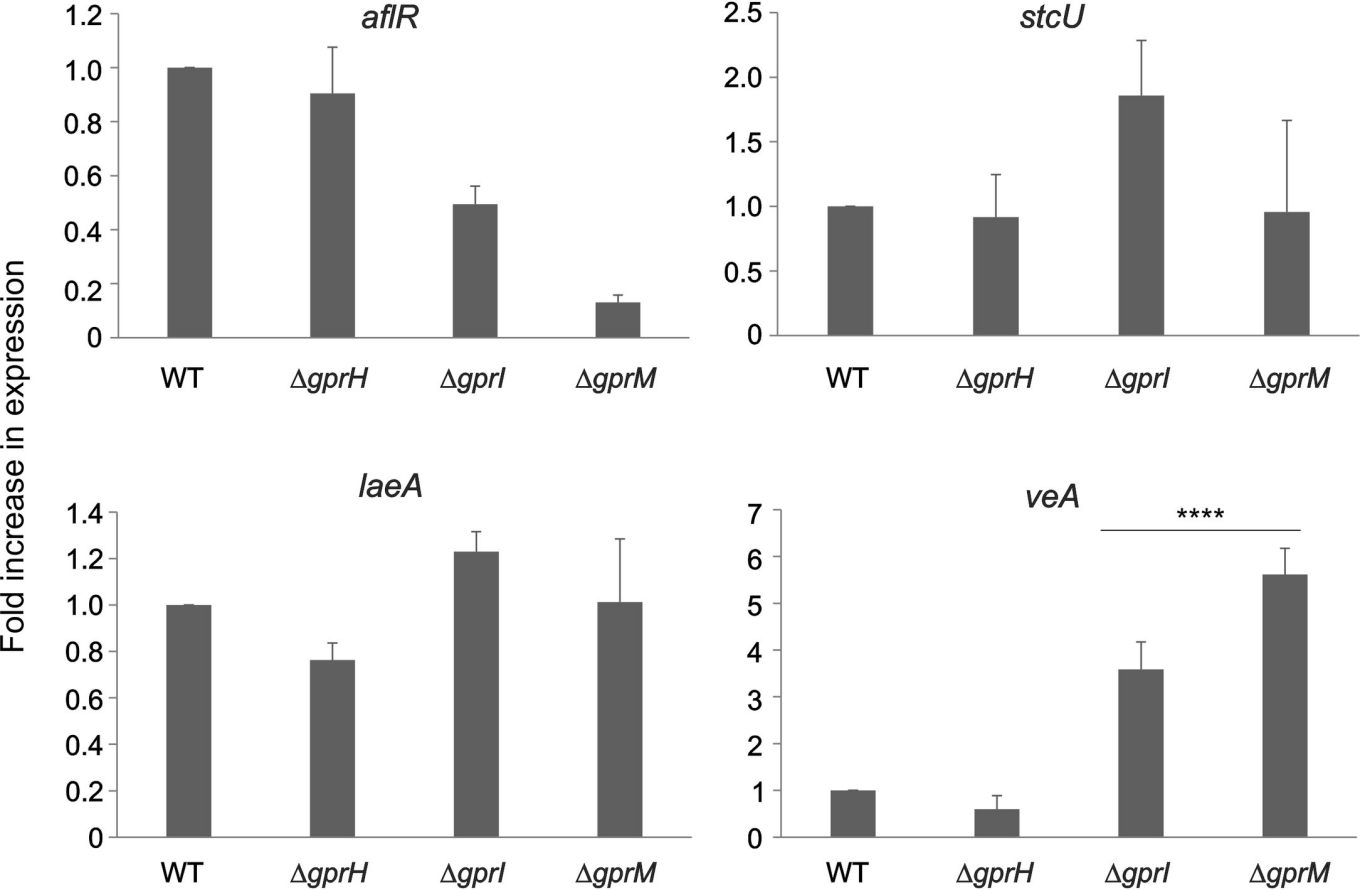
The effect of GPCRs on the transcriptional regulation of regulators on ST production. The fold change in expression levels of genes involved in ST biosynthesis (*aflR*, *veA*, *stcU* and *laeA*) compared to the wild-type strain when cultured in MM with glucose 1% (w/v) at 37°C for 72 h in the dark. Gene expression analysis was carried out using quantitative real-time PCR (RT-qPCR). The *A*. *nidulans tubC* gene was used as a reference for normalization. **** *p<0*.*0001*.

The influence of the three receptor on the transcriptional regulation of known regulators of ST production was assessed after 3 days cultivation under the same ST-inducing conditions. In the ⨂*gprM* mutant, the expression of the *aflR* transcription factor was reduced, while the expression of the ST biosynthetic enzyme encoding *stcU*, and the global *laeA* regulator, was comparable to the wild-type strain. However, the *veA* expression was significantly elevated in the ⨂*gprI* and ⨂*gprM* mutants (Fig 10). This suggests that the GprI and GprM pathway may influence *veA* transcriptionally, while the GprH pathway influences VeA nuclear localization and regulation, collectively altering VeA activity which increased ST production in the Δ*gprM* and Δ*gprH*Δ*gprM* mutants.

Since GprH, GprI and GprM differentially affected ST production, their influence on the production of another secondary metabolite, penicillin, was assessed using bacterial inhibition assays [48]. However, the absence of individual or multiple receptors did not influence the inhibition of bacterial growth and therefore penicillin production (Fig 9C) suggesting that these three GPCRs are dispensable for penicillin biosynthesis in *A*. *nidulans*.

## Discussion

Fungi sense extracellular signals to coordinate their development, and cell surface GPCRs coupled with G-protein signalling plays a crucial role in initiating this response [49]. *A*. *nidulans* possesses 86 putative GPCRs [10], but only a few have been functionally characterised. The *A*. *nidulans* class III putative carbon receptor, GprD, is structurally related to the *S*. *cerevisiae* Gpr1 glucose receptor, and functions upstream of cAMP-PKA pathway, regulating conidial germination and hyphal growth on glucose, while repressing sexual development [4, 50]. Gene expression analyses identified additional GPCRs, namely class V *gprH* and class VII *gprM*, to be increasingly transcribed during carbon starvation, a condition where GprD is not expressed. GprH was previously shown to be a putative glucose and amino acid receptor functioning upstream of the cAMP-PKA pathway [16, 38]. An additional class V receptor, gprI, remained uncharacterised. This led to the hypothesis that GprH, GprI and GprM may collectively represent a carbon starvation-induced nutrient sensing mechanism. Individually, the absence of a single receptor caused the over-expression of another receptor, suggesting the existence of redundant or compensatory functions among these carbon starvation-induced receptors. Subsequent genetic analyses demonstrated that GprH functioned in parallel to an epistatic interaction between GprI and GprM, in the regulation of fungal growth, light-dependent sexual development and ST mycotoxin production.

In fungi, GPCRs activate G-protein signalling which initiates the cAMP-PKA pathway [7]. cAMP levels are influenced by glucose availability and increase during glucose starvation. The reintroduction of glucose causes a cAMP burst, which activates PKA [51]. Here, the Δ*gprH*, Δ*gprI* and Δ*gprM* strains did not display increased cAMP levels during carbon starvation, or a burst of cAMP in response to glucose, suggesting that GprH, GprI and GprM all function upstream of the cAMP-PKA pathway. GprH positively influences conidial germination and glucose uptake [16]. Individually, the absence of GprI or GprM had no influence on germination, but the combined absence of both GprI and GprM delayed conidial germination, suggesting epistasis and/or functional redundancy. Intriguingly, the simultaneous absence of all three receptors restored germination defects. The individual Δ*gprH*, Δ*gprI* and Δ*gprM* null mutants all showed a reduction in hyphal growth after the starvation and re-introduction of glucose. In contrast, the combined absence of two or three receptors caused the hyper-activation of hyphal growth during carbon starvation. This correlated with the recovery of cAMP signalling in the double and triple null mutants. Therefore, collectively GprH, GprI and GprM, influence germination and hyphal growth post carbon starvation, but the absence of multiple receptors activates an unknown compensatory cAMP signalling mechanism to promote hyphal growth.

Pheromone sensing, nutrient availability and light determine if *A*. *nidulans* undergoes sexual or asexual reproduction [41]. Sexual development requires well-nourished, dark conditions [43]. The GprA/GprB pheromone signalling pathway promotes sexual development [15], while GprD acts as a repressor of sexual development [4], functioning upstream of the pheromone pathway [15]. Similarly, GprH also acts as a repressor of sexual development, but during carbon starvation. The absence of GprH altered the expression of regulators of sexual development, increasing *nsdD*, and reducing *nosA* and *rosA* transcription, resulting in increased cleistothecia production [16]. In contrast to Δ*gprH*, the Δ*gprI* and Δ*gprM* mutants did not show enhanced sexual development. However, the combined absence of any two, or all three, receptors resulted in the derepression of sexual development and enhanced cleistothecia production. Again, this provides evidence that GprH functioned in parallel to an epistatic interaction between GprI and GprM in the regulation of sexual development during carbon starvation.

VeA is a light-dependent activator of sexual development [17]. Glucose concentration also affects the light-dependent localization of the VeA transcription factor, as well as the functions of the white collar complex (LreA and LreB) plus the red-light FphA phytochrome [34]. Overexpression of *veA* causes the increased activation of sexual development [52, 53]. In the dark, VeA accumulates in the nucleus, but this is repressed by the absence of glucose, irrespective of the presence of the GprH receptor. However, during light exposed carbon starvation, the absence of GprH increased the accumulation of VeA in the nucleus, promoting sexual development [24, 26]. This effect on VeA subcellular localization is also reminiscent of that observed in the absence of FphA [18]. The derepression of sexual development caused by the absence of GprD or FphA only occurs in the *veA*^+^ background [4, 52]. Introducing the *veA1* mutation into the Δ*gprH* background abolished the derepression of sexual development, confirming that GprH controlled sexual development in a VeA-dependent manner during light exposed carbon starvation. Therefore, both GprH and GprD repress sexual development via regulating VeA function, but under different nutritional states. How these two carbon sensing mechanisms interact with VeA remains unknown.

VeA also interacts with the LaeA methyl transferase that regulates the expression of secondary metabolite gene clusters, while both VeA and LaeA are required for ST mycotoxin production [33, 35]. G-protein signalling similarly affects the ST production [54] and the downstream cAMP-PKA pathway represses the expression of the *aflR* transcriptional activator of the ST biosynthetic gene cluster, and consequently inhibits ST production [32]. This suggests that GprH, GprI and GprM may influence ST production in a light-dependent manner. The effect of light on fungal ST production also depends on the nutritional state, as by increasing glucose concentration it is possible to by-pass the partial repression of ST production caused by light [34]. VeA accumulates in the nucleus independent of light, according to increased glucose availability, and coinciding with an increased production of ST [34]. How the effects of light and glucose availability are integrated in the regulation of ST production remains unknown. Here, the three receptors differentially influenced ST production, where GprM alone had a major, and GprH a minor, role in the repression of ST production. Interestingly, the increased production of ST in the Δ*gprM and* Δ*gprH*Δ*gprM* mutants was dependent on GprI, as the triple Δ*gprH*Δ*gprI*Δ*gprM* mutant restored wild-type ST production. The transcriptional analysis of genes involved in regulating ST production revealed the GprI and GprM pathway to repress *veA* expression, which was in contrast to GprH which was shown to regulate VeA nuclear localisation. It remains unknown if GprI and GprM regulate VeA localisation, but this data suggests that these two GPCR mediated pathway may modulate VeA activity via distinct transcriptional and post-translational mechanisms.

In conclusion, important environmental factors such as light and carbon availability affect fungal development and secondary metabolism in *A*. *nidulans*. Here, we show how cellular responses to these stimuli occur in an interconnected manner. We revealed GprH, GprI and GprM to be a novel carbon starvation-induced glucose sensing system that functions upstream of the cAMP-PKA, which interacts with the VeA-mediated light sensing mechanism, to regulate fungal development and mycotoxin production. Genetic studies showed the GprH functions in parallel to the epistatic interaction between GprI and GprM in the regulation of sexual development and ST production. Protein-protein interactions studies are now required to dissect how these GPCRs, and their G-proteins [32, 55], interact with the light-sensing systems to control fungal development and ST production. These studies show the complex and integrated structure of fungal mechanisms that perceive different environmental stimuli, through multiple receptors, in turn coordinating fungal development and secondary metabolism, to achieve optimal survival when exposed to environmental versatility. Future studies may reveal how these environment-sensing mechanisms interact to enable fungi to adapt to a fluctuating environment.

## Materials and methods

### *Aspergillus nidulans* strains, construction of fungal mutants and growth conditions

Genotypes of the *A*. *nidulans* strains used are listed in Supplementary S1 Table and nomenclature of genes is described in [51]. AGB551 and TN02A3 were used as reference strains according to *veA*^*+*^ or *veA1* backgrounds, respectively. Null *gpr* strains were generated either by transformation with DNA replacement cassettes [56] or by crossing and selecting recombinants [57]. The *gprH*, *gprI* and *gprM* deletion cassettes were obtained following the fusion-PCR protocols described in [58]. All PCR reactions were performed using TaKaRa Ex Taq DNA Polymerase (ClontechUSA). Supplementary S2 Table lists the specific oligonucleotides used to generate each of the transformation cassettes. *Aspergillus fumigatus* (Af) *pyrG*^*Af*^ and *pyroA*^*Af*^ genes were used as prototrophic selection markers. To study the subcellular localization of the VeA::GFP fusion protein in the Δ*gprH* mutant, the TRMD3.4.17 strain containing VeA::GFP [17] was crossed with Δ*gprH* [16], generating the Δ*gprH VeA*::*GFP* strain. The corresponding recombinant strain was selected. To further generate the *VeA*::*GFP mRFP*::*h2A* and the *VeA*::*GFP* Δ*gprH mRFP*::*h2A* strains, the mRFP::h2A cassette was generated by *in vitro* recombination as described in [58] with the exception that genes were not replaced by prototrophic markers but were instead N-terminally tagged to mRFP. In this case, the *prtA* gene was used as a selective marker. Briefly the 5´-UTR and 3´-UTR sequence from histone 2A (h2A) was PCR amplified from genomic DNA from *A*. *nidulans* A4 (FGSC). The DNA sequence containing the mRFP plus the h2A gene and its terminator was PCR amplified. The pyrithiamine gene was amplified from pPTRI plasmid (TaKaRa Bio). All fragments were co-transformed, together with plasmid pRS426, previously linearized with *EcoR*I and *BamH*I, into *S*. *cerevisiae* SC9721 using the lithium acetate method [59]. *S*. *cerevisiae* was plated onto selective medium. The whole cassette containing h2A N-tagged to mRFP and the selective marker pyrithiamine was then PCR-amplified from *S*. *cerevisiae* genomic DNA, purified and used to transform *A*. *nidulans* TRMD3.4.17 or Δ*gprH VeA*::*GFP* strains, according to [60]. Transformed candidates were plated in selective medium containing pyrithiamine, purified and checked by both, PCR and microscopy.

To generate the triple null mutant Δ*gprH*Δ*gprI*Δ*gprM*, the double mutant Δ*gprH*Δ*gprI* was grown on a plate containing uracil, uridine and the mutagen 5-fluoroorotic acid (5-FOA) (0.75 mg/ml) that allows the recovery of pyrimidine auxotrophs through inactivation of the OMP decarboxylase gene (*pyrG*) [61]. After three days, sectors showing improved growth were isolated and purified on media containing uracil and uridine. One of the FOA resistant and *pyrG*^-^ strains was used as recipient of the cassette Δ*gprM*::*pyrG*^*Af*^. For both protoplast mediated transformation and sexual crosses, homologous recombination events were verified by Southern blotting (Supplementary S2 Fig).

To obtain the complemented strains Δ*gprH*::*gprH*^+^ (*veA+)* and Δ*gprI*::*gprI*^+^ (*veA+)*, the complementing cassettes containing the 5´-UTR region plus the *gprH* or *gprI* gene and the 3′-UTR sequences were PCR- amplified from *A*. *nidulans* genomic DNA. The auxotopyc marker pyrG was amplified from pCDA21 plasmid and inserted into the complementing cassete as a prototrophic marker. Again, the complementing cassetes were obtained by *in vivo* recombination in *S*. *cerevisiae*. After, the cassetes were fully amplified by PCR and transformed into the Δ*gprH* or Δ*gprI* null mutants (both *veA*^*+*^ strains). Positive *A*. *nidulans* complementing candidates were selected and purified through three rounds of growth on plates, gDNA was extracted, and the candidates were confirmed by PCR.

*Aspergillus* complete (YG) and minimal (MM) media, plus the required supplements, were used as described by [62], with or without the addition of 2% agar. Solid YG and MM supplemented for auxotrophies were used to produce conidia for propagation and the maintenance of strains. *A*. *nidulans* was grown at 37°C. Growth tests were performed in MM and colony growth was scored at 37°C for 48 h (under darkness or constant illumination).

### RNA extractions and analysis of gene expression

The expression levels of GPCR genes during carbon starvation was analysed as described by [16]. Briefly, 1x10^7^ conidia of the corresponding wild-type and mutant strains were inoculated in MM with glucose 1% (w/v) and incubated at 37°C for 24 h. Next, mycelia were washed and shifted to MM with no carbon source at 37°C for 4 or 8 h (short-term starvation). Samples at corresponding intervals were frozen in liquid nitrogen and total RNA was extracted using Triazol (Invitrogen) reagent and purified using RNeasy Plant Mini Kit (Quiagen). cDNA was synthesised from 5 μg of RNA using SuperScript III (Invitrogen). Gene expression analysis was carried out using quantitative real-time PCR (RT-qPCR) according to [63]. cDNA of *A*. *nidulans tubC* gene was used for normalization. Corresponding primers for *gprH*, *gprI* and *gprM* are listed in Supplementary S2 Table.

The expression levels of genes involved in ST biosynthesis (*aflR*, *veA*, *stcU* and *laeA*), was performed as followed. Briefly, 1x10^6^ conidia/mL of the corresponding wild-type and mutant strains were inoculated in MM with glucose 1% (w/v) and incubated at 37°C, 180rpm, for 72 h under dark condition. Mycelia was filtered and frozen in liquid nitrogen. Total RNA was extracted using Triazol (Invitrogen) reagent and purified using RNeasy Plant Mini Kit (Quiagen). cDNA was synthesised from 5 μg of RNA using SuperScript III (Invitrogen). Gene expression analysis was carried out using quantitative real-time PCR (RT-qPCR) according to [64]. Primers used for the RT-qPCR analysis are listed in Supplementary S2 Table. The *A*. *nidulans* tubC gene was used as a reference.

### cAMP quantification

1x10^7^ conidia of the AGB551 wild-type and corresponding mutant strains were inoculated in liquid YG media and incubated at 37°C and 180 rpm for 16 h. Next, mycelia were washed and then incubated in MM lacking a carbon source, at 37°C for 4 h. After this time, glucose at final concentration of 2% (w/v) was added and incubated for 0.5, 1, 3, 5 or 10 mins. Mycelia were used for cAMP extractions according to (16). The cAMP Biotrak EIA system assays (GE Healthcare) were performed according to manufacturer’s instructions. Total protein content in the corresponding extracts was measured using the Bio-Rad protein assay following manufacturer's instructions. cAMP concentration was obtained using the ELISA software and data analysis (http://elisaanalysis.com/) and are presented as fmol per μg total protein.

### Microscopy

To analyse the growth of germlings post-carbon starvation, 1x10^5^ conidia of the *A*. *nidulans* strains were incubated in MM with glucose 2% (w/v) for 3 h at 37°C and then transferred to the modified MM with no carbon and nitrogen sources for 3 h. Next, the starved germlings were shifted to MM for an additional 1 h. Cells were fixed (3% (v/v) formaldehyde and 1.5% (v/v) DMSO in 1× PBS (140 mM NaCl, 2 mM KCl, 10 mM NaHPO_4_, 1.8 mM KH_2_PO_4_, pH 7.4) at room temperature for 10 min and then washed in PBS. For germination assays, 1x10^5^ conidia of the corresponding strains were incubated in MM with glucose 1% for 6 h at 37°C. After this time, cells were fixed and washed in PBS. All samples were observed using a Zeiss epifluorescence microscope, and the phase contrast bright-field images were captured with AxioCam camera and processed using the AXIOVISION software version 3.1 (Carl Zeiss).

To study the subcellular localization of the VeA::GFP fusion protein, conidia of the wild-type TRMD3.4.17 and the Δ*gprH*:VeA::GFP mutant strains were germinated in MM (glucose 1%) and further incubated for 14 h at 25°C, with white light (28 microEinsteins/m^2^/s) or in the dark. Next, the cells were shifted to MM with no carbon source for 3 h (light or dark). Samples were observed at 30 min, 1 h and 3 h of carbon starvation. Nuclei were stained with 12 μg/ml Hoechst (Life Technologies, Inc.) to verify the VeA::GFP nuclear localization. DIC images were taken with an exposure of 40 ms, DAPI with 150 ms and GFP with 1000 ms. Images were processed minimally using Wasabi (Hammamatsu Photonics) and ImageJ 1.37v software package.

### Quantification of sexual and asexual development

To assess cleistothecia production in liquid media, 1x10^7^ conidia of the AGB551, TN02A3 and all the null *gpr* strains were inoculated in 30 ml of MM with glucose 2% (w/v) and incubated at 37°C for 24 h and 180 rpm. Next, mycelia were filtered and transferred to MM containing no carbon source for 8 days, after which fruiting body production was assessed. Cultures were either exposed to white light (28 microEinsteins/m^2^/s) or kept in the dark.

Cleistothecia and conidia production was quantified on solid media according to [64] with some modifications. 100 μl of a 1x10^6^ conidia/ml stock were plated MM and incubated at 37°C for 24 h in darkness. Next, one half of the plates was transferred to constant white light for 48 h (asexual) or 96 h (sexual), and the other one was kept in darkness for the same time periods. Plates assessing sexual development quantification were sealed after first 24 h incubation in darkness. To determine the amount of cleistothecia, a defined area of 3.14 mm^2^ was isolated and cleistothecia counted. For the quantification of conidiospores, they were collected in a 2% Tween20 solution and conidia/cm^2^ determined using a Neubauer chamber.

### Sterigmatocystin (ST) analysis by thin layer chromatography (TLC)

TLC analyses were carried using procedures previously utilized in [65] with minor modifications. Briefly, *A*. *nidulans* strains were inoculated (1x10^6^ conidia/ml) into 25 ml of MM with glucose 1% (w/v) liquid stationary cultures and incubated at 37°C in the dark for 7 days. ST was extracted with chloroform using a 1:1 ratio. Extracts were dried overnight and resuspended in 250 μl of chloroform. Samples were fractionated by TLC on Silica Pre-Coated Polygram Sil G/UV_254_ TLC plates (Macherey-Nagal using benzene and glacial acetic acid [95:5 (v/v)] or toluene:ethyl acetate:glacial acetic acid [80:10:10 (v/v/v)] as solvent systems. Plates were sprayed with aluminium chloride (15% in ethanol) and baked at 80°C for 10 min. Metabolites present on TLC plates were visualized under 375 nm UV light. Commercial ST (Sigma-Aldrich) was used as standard. Densitometry of TLC bands was carried out using Gelquant.NET software.

### Penicillin (PN) bioassay

The PN bioassay was performed as previously described [48] with some modifications, using *Bacillus calidolactis C953* as the test organism. Briefly, *A*. *nidulans* strains were inoculated (1x10^6^ conidia/ml) in 20 ml of a seed culture medium, and incubated at 200 rpm for 48 h. After incubation, mycelia were collected using sterile Miracloth (EDM Millipore-Calbiochem) and 1 g of mycelium from each fungal strain was inoculated in 20 ml of fermentation medium. The cultures were incubated at 26°C for 96 h at 250 rpm. After incubation, the cultures were filtered through Miracloth. 300 Ml of tryptone-soy agar was supplemented with 50 ml of *B*. *calidolactis* C953 culture and plated on five 150-mm-diameter Petri dishes. 10 μl of each culture supernatant was added to 7-mm-diameter wells. The plates were incubated at 55°C for 16 h, and the zones of inhibition (halos) were measured. To confirm that the observed antibacterial activity was due to the presence of PN and not to the presence of other fungal compounds in the supernatant, controls containing commercial penicillinase from *Bacillus cereus* (Sigma-Aldrich) were used.

### Nuclei protein extraction and Western blot analysis

Nuclear proteins were obtained according to Palmer and colleagues [66] with modifications. Briefly, 1 x 10^6^ conidia/ml of the *h2A*::*mRFP;VeA*::*GFP* (WT) or Δ*gprH;h2A*::*mRFP;VeA*::*GFP* strains were inoculated in 500 ml of liquid MM and incubated at 37°C for 16h (with or without light). Mycelia were filtered, washed three times with sterile water and transferred to MM without carbon source for 3h at 37°C (with or without light). Mycelia were filtered, frozen in liquid nitrogen, and ground to powder. Powdered mycelia were suspended in 20 ml nuclei isolation buffer- NIB (1 M sorbitol, 10 mM Tris-HCl, pH 7.5, 0.15 mM spermine, 0.5 mM spermidine, 10 mM EDTA, 2.5 mM phenylmethylsulfonyl fluoride, 1mM DTT, 1 pill/10ml EDTA-free complete Mini protease inhibitor [Roche]) on ice. Samples were centrifuged 10min, 1000 x *g* at 4°C. Supernatant was filtered through two layers of Miracloth and centrifuged at 10,000 x *g* for 15 min at 4°C. The pellet was resuspended in 1.5 ml resuspension buffer- RB (1 M sorbitol, 10 mM Tris-HCl, pH 7.5, 0.15 mM spermine, 0.5 mM spermidine, 1 mM EDTA, 2.5 mM phenylmethylsulfonyl fluoride, 1mM DTT, 1 pill/10ml EDTA-free complete Mini protease inhibitor [Roche]) and then centrifuged at 12,000 rpm, 15min at 4°C. Supernatant was removed and crude nuclei were suspended in 0.4 mL of a modified ST buffer (1 M sorbitol, 10 mM Tris-HCl, pH 7.5, 0.5% NP40, 0.1 mM DTT, 1 pill/10 ml EDTA-free complete Mini protease inhibitor [Roche]). Cellular debris were pelleted by centrifugation (4800 rpm, 30s at 4°C) and the protein content was measured using a Bradford assay (BioRad).

For each nuclear protein sample, a total of 20μl of GFP by Trap Agarose (ChromoTek) was washed three times with cold ST buffer and incubated for 4h with of 500 μg of nuclear protein extract with shaking at 4°C. Further, the samples were centrifuged at 5000 x *g* for 30 seconds and the resin was washed twice with cold ST buffer at 4°C. Dissociation of bound GFP proteins was performed through addition of SDS-sample buffer and boiling at 95°C for 5min. Samples were loaded on a 10% SDS-PAGE gel and transferred onto a nitrocellulose membrane using the iBlot 2 Dry Blotting System (Life Technologies) according to the manufacturer’s instructions. For mRFP blotting, a total of 20μg of nuclear protein of each sample was loaded on a 10% SDS-PAGE gel and similarly transferred onto a nitrocellulose membrane. After protein transference, all membranes were blocked for 1 h in 5% (w/v) dry skimmed milk in TBS-T [0.14 M NaCl, 0.02 M Tris, and 0.1% (v/v) Tween 20, pH 7.6] at room temperature. Following, membranes were washed with TBS-T and incubated with a 1:1000 dilution of primary antibody anti-GFP (Sant-Cruz Biotechnology, Inc: Sc-9996) or anti-RFP (ABcam: AB65856) overnight at 4°C. Primary antibodies were detected using a dilution of 1:10,000 of a horseradish peroxidase (HRP)-conjugated second antibody (Cell Signaling Technology) at room temperature for 1h. Membranes were washed three times with TBS-T and revealed using ECL plus Western Blotting Detection Reagents (Amersham). Coomassie stained gels containing 20 μg of nuclear protein confirmed protein loading.

## Supporting information

S1 Fig The corrected *gprI* (AN8348_M) gene model. A) BLAT alignment of the gene AN8348 on the sense strand of chromosome V of the *A*. *nidulans* FGSC_A4 genome shows only all other *Aspergillus* species and closely related fungi to present an alternative gene model on the antisense strand. B) An alignment of AN8348 with the modified gene model AN8348_M showing their differing orientation, overlap, and intro/exon boundaries. C) InterPro analysis of the AN8348_M gene model yields 7-TM domain containing GPCR, with the Dicty_CAR domain characteristic of class V fungal GPCRs.
(TIF)

S2 Fig Southern Blot analysis confirms deletion of the different *A*. *nidulans* GPCRs. A) Deletion of *gprI* in the Δ*gprI* and Δ*gprI*Δ*gprM* strains. B) Deletion of *gprI* in the Δ*gprH*Δ*gprI* strain. C) Deletion of *gprM* in the Δ*gprM*, Δ*gprH*Δ*gprM* and Δ*gprH*Δ*gprI*Δ*gprM* strains.
(TIF)

S1 Table List of *A*. *nidulans* strains used in this work. (DOCX)

S2 Table List of primers used in this work. (DOCX)

S1 File All the raw data presented in the Figs 1–10. (XLSX)

## References

[1] Morris AJ, Malbon CC. Physiological regulation of G protein-linked signaling. Physiol Rev. 1999;79(4):1373–430. 10.1152/physrev.1999.79.4.1373 10508237

[2] Neves SR, Ram PT, Iyengar R. G protein pathways. Science. 2002;296(5573):1636–9. 10.1126/science.1071550 12040175

[3] Bolker M. Sex and crime: heterotrimeric G proteins in fungal mating and pathogenesis. Fungal Genet Biol. 1998;25(3):143–56. 10.1006/fgbi.1998.1102 9917369

[4] Han KH, Seo JA, Yu JH. A putative G protein-coupled receptor negatively controls sexual development in *Aspergillus nidulans*. Mol Microbiol. 2004;51(5):1333–45. 10.1111/j.1365-2958.2003.03940.x 14982628

[5] Lee N, D'Souza CA, Kronstad JW. Of smuts, blasts, mildews, and blights: cAMP signaling in phytopathogenic fungi. Annu Rev Phytopathol. 2003;41:399–427. 10.1146/annurev.phyto.41.052002.095728 12651963

[6] Lengeler KB, Davidson RC, D'souza C, Harashima T, Shen WC, Wang P, et al Signal transduction cascades regulating fungal development and virulence. Microbiol Mol Biol Rev. 2000;64(4):746–85. 10.1128/mmbr.64.4.746-785.2000 11104818 PMC99013

[7] Yu JH, Keller N. Regulation of secondary metabolism in filamentous fungi. Annu Rev Phytopathol. 2005;43:437–58. 10.1146/annurev.phyto.43.040204.140214 16078891

[8] Pontecorvo G, Roper JA, Hemmons LM, MacDonald KD, Bufton AW. The genetics of *Aspergillus nidulans*. Adv Genet. 1953;5:141–238. 13040135 10.1016/s0065-2660(08)60408-3

[9] Paulussen C, Hallsworth JE, Alvarez-Perez S, Nierman WC, Hamill PG, Blain D, et al Ecology of aspergillosis: insights into the pathogenic potency of *Aspergillus fumigatus* and some other *Aspergillus* species. Microb Biotechnol. 2017;10(2):296–322. 10.1111/1751-7915.12367 27273822 PMC5328810

[10] Li L, Wright SJ, Krystofova S, Park G, Borkovich KA. Heterotrimeric G protein signaling in filamentous fungi. Annu Rev Microbiol. 2007;61:423–52. 10.1146/annurev.micro.61.080706.093432 17506673

[11] Brown NA, Schrevens S, van Dijck P, Goldman GH. Fungal G-protein-coupled receptors: mediators of pathogenesis and targets for disease control. Nature Microbiology. 2018;3(4):402–14. 10.1038/s41564-018-0127-5 29588541

[12] DeZwaan TM, Carroll AM, Valent B, Sweigard JA. *Magnaporthe grisea* Pth11p is a novel plasma membrane protein that mediates appressorium differentiation in response to inductive substrate cues. Plant Cell. 1999;11(10):2013–30. 10.1105/tpc.11.10.2013 10521529 PMC144101

[13] Dilks T, Halsey K, De Vos RP, Hammond-Kosack KE, Brown NA. Non-canonical fungal G-protein coupled receptors promote Fusarium head blight on wheat. Plos Pathogens. 2019;15(4). 10.1371/journal.ppat.1007666 PMC6459559 30934025

[14] Hoffmann B, Wanke C, Lapaglia SK, Braus GH. c-Jun and RACK1 homologues regulate a control point for sexual development in *Aspergillus nidulans*. Mol Microbiol. 2000;37(1):28–41. 10.1046/j.1365-2958.2000.01954.x 10931303

[15] Seo JA, Han KH, Yu JH. The *gprA* and *gprB* genes encode putative G protein-coupled receptors required for self-fertilization in *Aspergillus nidulans*. Mol Microbiol. 2004;53(6):1611–23. 10.1111/j.1365-2958.2004.04232.x 15341643

[16] Brown NA, Dos Reis TF, Ries LN, Caldana C, Mah JH, Yu JH, et al G-protein coupled receptor-mediated nutrient sensing and developmental control in *Aspergillus nidulans*. Mol Microbiol. 2015;98(3):420–39. 10.1111/mmi.13135 26179439

[17] Stinnett SM, Espeso EA, Cobeno L, Araujo-Bazan L, Calvo AM. *Aspergillus nidulans* VeA subcellular localization is dependent on the importin alpha carrier and on light. Mol Microbiol. 2007;63(1):242–55. 10.1111/j.1365-2958.2006.05506.x 17163983

[18] Purschwitz J, Muller S, Kastner C, Schoser M, Haas H, Espeso EA, et al Functional and physical interaction of blue- and red-light sensors in *Aspergillus nidulans*. Curr Biol. 2008;18(4):255–9. 10.1016/j.cub.2008.01.061 18291652

[19] Bayram O, Braus GH, Fischer R, Rodriguez-Romero J. Spotlight on *Aspergillus nidulans* photosensory systems. Fungal Genet Biol. 2010;47(11):900–8. 10.1016/j.fgb.2010.05.008 20573560

[20] Calvo AM, Lohmar JM, Ibarra B, Satterlee T. Velvet regulation of fungal development. J Wedland. Springer International Publishing.; 2016 p. 475–97.

[21] Mooney JL, Hassett DE, Yager LN. Genetic analysis of suppressors of the *veA1* mutation in *Aspergillus nidulans*. Genetics. 1990;126(4):869–74. 2076818 10.1093/genetics/126.4.869 PMC1204284

[22] Mooney JL, Yager LN. Light is required for conidiation in *Aspergillus nidulans*. Genes Dev. 1990;4(9):1473–82. 10.1101/gad.4.9.1473 2253875

[23] Dasgupta A, Fuller KK, Dunlap JC, Loros JJ. Seeing the world differently: variability in the photosensory mechanisms of two model fungi. Environ Microbiol. 2016;18(1):5–20. 10.1111/1462-2920.13055 26373782 PMC4757429

[24] Bayram O, Krappmann S, Ni M, Bok JW, Helmstaedt K, Valerius O, et al VelB/VeA/LaeA complex coordinates light signal with fungal development and secondary metabolism. Science. 2008;320(5882):1504–6. 10.1126/science.1155888 18556559

[25] Bayram O, Braus GH. Coordination of secondary metabolism and development in fungi: the velvet family of regulatory proteins. FEMS Microbiol Rev. 2012;36(1):1–24. 10.1111/j.1574-6976.2011.00285.x 21658084

[26] Calvo AM, Lohmar JM, Ibarra B, Satterlee T. Velvet Regulation of Fungal Development In: Wendland J, editor. Growth, Differentiation and Sexuality, 3rd Edition Mycota-A Comprehensive Treatise on Fungi as Experimental Systems for Basic and Applied Research 12016. p. 475–97.

[27] Calvo AM, Wilson RA, Bok JW, Keller NP. Relationship between secondary metabolism and fungal development. Microbiol Mol Biol Rev. 2002;66(3):447–59, table. 10.1128/MMBR.66.3.447-459.2002 12208999 PMC120793

[28] Yu JH, Leonard TJ. Sterigmatocystin biosynthesis in *Aspergillus nidulans* requires a novel type I polyketide synthase. J Bacteriol. 1995;177(16):4792–800. 10.1128/jb.177.16.4792-4800.1995 7642507 PMC177246

[29] Wu F, Groopman JD, Pestka JJ. Public health impacts of foodborne mycotoxins. Annu Rev Food Sci Technol. 2014;5:351–72. 10.1146/annurev-food-030713-092431 24422587

[30] Jiang X, Wang J, Xing L, Shen H, Lian W, Yi L, et al Sterigmatocystin-induced checkpoint adaptation depends on Chk1 in immortalized human gastric epithelial cells in vitro. Arch Toxicol. 2017;91(1):259–70. 10.1007/s00204-016-1682-2 26914363

[31] Fernandes M, Keller NP, Adams TH. Sequence-specific binding by *Aspergillus nidulans* AflR, a C6 zinc cluster protein regulating mycotoxin biosynthesis. Mol Microbiol. 1998;28(6):1355–65. 10.1046/j.1365-2958.1998.00907.x 9680223

[32] Shimizu K, Keller NP. Genetic involvement of a cAMP-dependent protein kinase in a G protein signaling pathway regulating morphological and chemical transitions in *Aspergillus nidulans*. Genetics. 2001;157(2):591–600. 11156981 10.1093/genetics/157.2.591 PMC1461531

[33] Bok JW, Keller NP. LaeA, a regulator of secondary metabolism in *Aspergillus spp*. Eukaryot Cell. 2004;3(2):527–35. 10.1128/EC.3.2.527-535.2004 15075281 PMC387652

[34] Atoui A, Kastner C, Larey CM, Thokala R, Etxebeste O, Espeso EA, et al Cross-talk between light and glucose regulation controls toxin production and morphogenesis in *Aspergillus nidulans*. Fungal Genet Biol. 2010;47(12):962–72. 10.1016/j.fgb.2010.08.007 20816830 PMC2982864

[35] Kato N, Brooks W, Calvo AM. The expression of sterigmatocystin and penicillin genes in *Aspergillus nidulans* is controlled by *veA*, a gene required for sexual development. Eukaryot Cell. 2003;2(6):1178–86. 10.1128/EC.2.6.1178-1186.2003 14665453 PMC326641

[36] Bayram O, Bayram OS, Ahmed YL, Maruyama J, Valerius O, Rizzoli SO, et al The *Aspergillus nidulans* MAPK module AnSte11-Ste50-Ste7-Fus3 controls development and secondary metabolism. PLoS Genet. 2012;8(7):e1002816 10.1371/journal.pgen.1002816 22829779 PMC3400554

[37] Klein PS, Sun TJ, Saxe CL, III, Kimmel AR, Johnson RL, Devreotes PN. A chemoattractant receptor controls development in *Dictyostelium discoideum*. Science. 1988;241(4872):1467–72. 10.1126/science.3047871 3047871

[38] Krohn NG, Brown NA, Colabardini AC, Reis T, Savoldi M, Dinamarco TM, et al The *Aspergillus nidulans* ATM kinase regulates mitochondrial function, glucose uptake and the carbon starvation response. G3 (Bethesda). 2014;4(1):49–62. 24192833 10.1534/g3.113.008607 PMC3887539

[39] Stajich JE, Harris T, Brunk BP, Brestelli J, Fischer S, Harb OS, et al FungiDB: an integrated functional genomics database for fungi. Nucleic Acids Research. 2012;40(D1):D675–D81. 22064857 10.1093/nar/gkr918 PMC3245123

[40] Ni M, Rierson S, Seo JA, Yu JH. The *pkaB* gene encoding the secondary protein kinase A catalytic subunit has a synthetic lethal interaction with *pkaA* and plays overlapping and opposite roles in *Aspergillus nidulans*. Eukaryot Cell. 2005;4(8):1465–76. 10.1128/EC.4.8.1465-1476.2005 16087751 PMC1214532

[41] Han KH, Lee DB, Kim JH, Kim MS, Han KY, Kim WS, et al Environmental factors affecting development of *Aspergillus nidulans*. J Microbiol. 2003;41:34–40.

[42] Han DM, Han YJ, Chae KS, Jahng KY, Y.H. L. Effects of various carbon sources on the development of *Aspergillus nidulans* with *velA*~ or *velAl* allele. Kor J Mycol. 1994;22:332.

[43] Krijgsheld P, Bleichrodt R, van Veluw GJ, Wang F, Muller WH, Dijksterhuis J, et al Development in *Aspergillus*. Stud Mycol. 2013;74(1):1–29. 10.3114/sim0006 23450714 PMC3563288

[44] Yager LN. Early developmental events during asexual and sexual sporulation in *Aspergillus nidulans*. Biotechnology. 1992;23:19–41. 1504597

[45] Palmer JM, Theisen JM, Duran RM, Grayburn WS, Calvo AM, Keller NP. Secondary metabolism and development is mediated by LlmF control of VeA subcellular localization in *Aspergillus nidulans*. PLoS Genet. 2013;9(1):e1003193 10.1371/journal.pgen.1003193 23341778 PMC3547832

[46] Champe SP, M.B. Kurtz, L.N. Yager, N.J. Butnick, D.E. Axelrod. Spore formation in *Aspergillus nidulans*: Competence and other developmental processes The fungal spore: Morphogenetic controls,. New York, N.Y., USA: Academic Press, Inc; 1981 p. 63–91.

[47] Calvo AM. The VeA regulatory system and its role in morphological and chemical development in fungi. Fungal Genet Biol. 2008;45(7):1053–61. 10.1016/j.fgb.2008.03.014 18457967

[48] Brakhage AA, Browne P, Turner G. Analysis of the regulation of penicillin biosynthesis in *Aspergillus nidulans* by targeted disruption of the *acvA* gene. Mol Gen Genet. 1994;242(1):57–64. 10.1007/bf00277348 8277946

[49] Van Dijck P, Brown NA, Goldman GH, Rutherford J, Xue CY, Van Zeebroeck G. Nutrient Sensing at the Plasma Membrane of Fungal Cells. Microbiology Spectrum. 2017;5(2). 10.1128/microbiolspec.funk-0031-2016 PMC11687466 28256189

[50] de Souza WR, Morais ER, Krohn NG, Savoldi M, Goldman MHS, Rodrigues F, et al Identification of metabolic pathways influenced by the G-Protein coupled receptors GprB and GprD in *Aspergillus nidulans*. PLoS One. 2013;8:1–13. 10.1371/journal.pone.0062088 PMC3641053 23658706

[51] Pawson T, Scott JD. Protein phosphorylation in signaling-50 years and counting. Trends Biochem Sci 30: 286–290. 10.1016/j.tibs.2005.04.013 15950870

[52] Blumenstein A, Vienken K, Tasler R, Purschwitz J, Veith D, Frankenberg-Dinkel N, et al The *Aspergillus nidulans* phytochrome FphA represses sexual development in red light. Curr Biol. 2005;15(20):1833–8. 10.1016/j.cub.2005.08.061 16243030

[53] Kim H, Han K, Kim K, Han D, Jahng K, Chae K. The *veA* gene activates sexual development in *Aspergillus nidulans*. Fungal Genet Biol. 2002;37(1):72–80. 12223191 10.1016/s1087-1845(02)00029-4

[54] Tag A, Hicks J, Garifullina G, Ake C Jr., Phillips TD, Beremand M, et al G-protein signalling mediates differential production of toxic secondary metabolites. Mol Microbiol. 2000;38(3):658–65. 10.1046/j.1365-2958.2000.02166.x 11069688

[55] Seo JA, Yu JH. The phosducin-like protein PhnA is required for Gbetagamma-mediated signaling for vegetative growth, developmental control, and toxin biosynthesis in *Aspergillus nidulans*. Eukaryot Cell. 2006;5(2):400–10. 10.1128/EC.5.2.400-410.2006 16467480 PMC1405901

[56] Tilburn J, Scazzocchio C, Taylor GG, Zabicky-Zissman JH, Lockington RA, Davies RW. Transformation by integration in *Aspergillus nidulans*. Gene. 1983;26(2–3):205–21. 10.1016/0378-1119(83)90191-9 6368319

[57] Todd RB, Davis MA, Hynes MJ. Genetic manipulation of *Aspergillus nidulans*: meiotic progeny for genetic analysis and strain construction. Nat Protoc. 2007;2(4):811–21. 10.1038/nprot.2007.112 17446881

[58] Nayak T, Szewczyk E, Oakley CE, Osmani A, Ukil L, Murray SL, et al A versatile and efficient gene-targeting system for *Aspergillus nidulans*. Genetics. 2006;172(3):1557–66. 10.1534/genetics.105.052563 16387870 PMC1456264

[59] Schiestl RH, Gietz RD. High-efficiency transformation of intact yeast-cells using single stranded nucleic-acids as a carrier. Current Genetics. 1989;16(5–6):339–46. 10.1007/bf00340712 2692852

[60] Osmani SA, May GS, Morris NR. Regulation of the messenger-RNA levels of *nimA*, a gene required for the G2-m transition in *Aspergillus nidulans*. Journal of Cell Biology. 1987;104(6):1495–504. 10.1083/jcb.104.6.1495 3294854 PMC2114495

[61] Boeke JD, LaCroute F, Fink GR. A positive selection for mutants lacking orotidine-5'-phosphate decarboxylase activity in yeast: 5-fluoro-orotic acid resistance. Mol Gen Genet. 1984;197(2):345–6. 10.1007/bf00330984 6394957

[62] de Assis LJ, Ries LN, Savoldi M, Dos Reis TF, Brown NA, Goldman GH. Aspergillus nidulans protein kinase A plays an important role in cellulase production. Biotechnol Biofuels. 2015;8:213 10.1186/s13068-015-0401-1 26690721 PMC4683954

[63] Semighini CP, Marins M, Goldman MH, Goldman GH. Quantitative analysis of the relative transcript levels of ABC transporter Atr genes in *Aspergillus nidulans* by real-time reverse transcription-PCR assay. Appl Environ Microbiol. 2002;68(3):1351–7. 10.1128/AEM.68.3.1351-1357.2002 11872487 PMC123782

[64] Rauscher S, Pacher S, Hedtke M, Kniemeyer O, Fischer R. A phosphorylation code of the Aspergillus nidulans global regulator VelvetA (VeA) determines specific functions. Molecular Microbiology. 2016;99(5):909–24. 10.1111/mmi.13275 26564476

[65] Ramamoorthy V, Dhingra S, Kincaid A, Shantappa S, Feng X, Calvo AM. The putative C2H2 transcription factor MtfA is a novel regulator of secondary metabolism and morphogenesis in *Aspergillus nidulans*. PLoS One. 2013;8(9):e74122 10.1371/journal.pone.0074122 24066102 PMC3774644

[66] Palmer JM, Perrin RM, Dagenais TRT, Keller NP. H3K9 Methylation Regulates Growth and Development in Aspergillus fumigatus. Eukaryotic Cell. 2008;7(12):2052–60. 10.1128/EC.00224-08 18849468 PMC2593193

