# Supplementary figures and images for: GPCR-mediated glucose sensing system regulates light-dependent fungal development and mycotoxin production
Source: PLoS Genet. 2019 Oct 14;15(10):e1008419. doi: 10.1371/journal.pgen.1008419 (PMC6812930; doi:10.1371/journal.pgen.1008419)

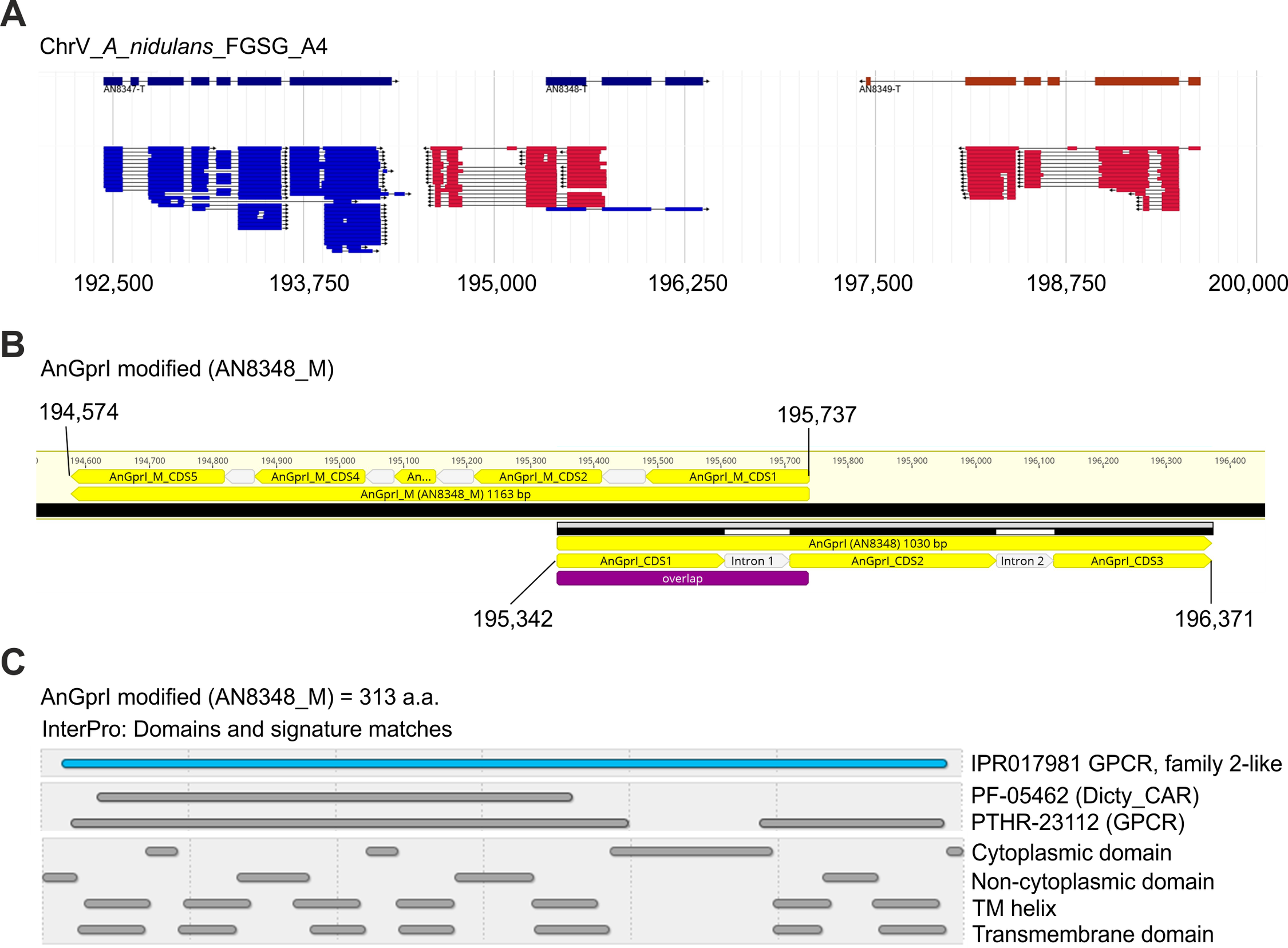

Supplement: S1 Fig — A) BLAT alignment of the gene AN8348 on the sense strand of chromosome V of the A. nidulans FGSC_A4 genome shows only all other Aspergillus species and closely related fungi to present an alternative gene model on the antisense strand. B) An alignment of AN8348 with the modified gene model AN8348_M showing their differing orientation, overlap, and intro/exon boundaries. C) InterPro analysis of the AN8348_M gene model yields 7-TM domain containing GPCR, with the Dicty_CAR domain characteristic of class V fungal GPCRs. (TIF) [file pgen.1008419.s001.tif]

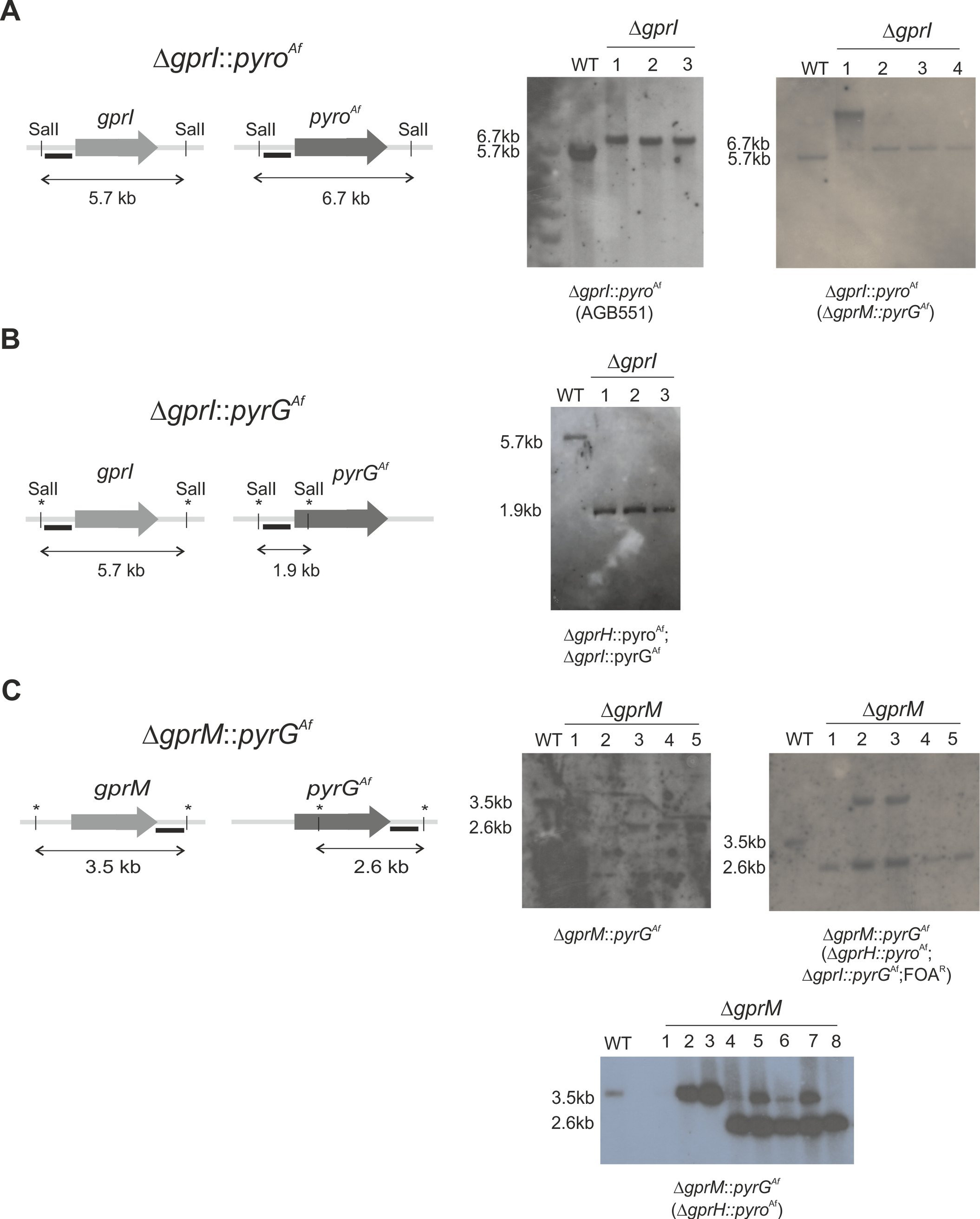

Supplement: S2 Fig — A) Deletion of gprI in the ΔgprI and ΔgprIΔgprM strains. B) Deletion of gprI in the ΔgprHΔgprI strain. C) Deletion of gprM in the ΔgprM, ΔgprHΔgprM and ΔgprHΔgprIΔgprM strains. (TIF) [file pgen.1008419.s002.tif]
