# Supplementary material for: GPCR-mediated glucose sensing system regulates light-dependent fungal development and mycotoxin production
Source: PLoS Genet. 2019 Oct 14;15(10):e1008419. doi: 10.1371/journal.pgen.1008419 (PMC6812930; doi:10.1371/journal.pgen.1008419)
Supplement: S1 Table — (DOCX) [file pgen.1008419.s003.docx]

Table S1. List of *A. nidulans* strains used in this work

| **Strain** | **Genotype** | **Reference** |
| --- | --- | --- |
| **AGB551** | *pyrG89, pyroA4, ΔnkuA::argB (argB2)* | (36) |
| **TNO2A3** | *pyrG89, pyroA4, ΔnkuA::argB (argB2), veA1* | (58) |
| **Δ*gprH* (*veA+*) ** | *pyrG89, pyroA4,ΔgprH::pyro^Af^, ΔnkuA::argB (argB2)* | (16) |
| **Δ*gprH* (*veA1*)** | *pyrG89, pyroA4,ΔgprH::pyro^Af^, ΔnkuA::argB (argB2), veA1* | This work |
| **Δ*gprH::gprH****^+^*** (*veA+*) ** | *pyrG89, pyroA4,ΔgprH::pyro^Af^,*  *gprH::pyrG^Af^*, *ΔnkuA::argB (argB2), VeA+* | This work |
| **Δ*gprI* (*veA+*) ** | *pyrG89, pyroA4, ΔgprI::pyro^Af^, ΔnkuA::argB (argB2)* | This work |
| **Δ*gprI::gprI****^+^***(*veA+*)** | *pyrG89, pyroA4,ΔgprI::pyro^Af^,*   *gprI^+^ ::pyrG^Af^*, *ΔnkuA::argB (argB2), VeA+* | This work |
| **Δ*gprM* ** | *pyrG89, pyroA4, ΔgprM::pyrG^Af^, nkuAΔ::argB (argB2)* | This work |
| **Δ*gprM::gprM****^+^*** (*veA+*)** | *pyrG89, pyroA4,ΔgprI::pyro^Af^,*  *gprM^+^*, *ΔnkuA::argB (argB2), VeA+, pyrithiamine resistant* | This work |
| **Δ*gprH*Δ*gprM*** | *pyrG89*, Δ*gprM*::*pyrG^Af^*, Δ*gprH*::*pyro^Af^, pyroA4*, Δ*nkuA::argB (argB2)* | This work |
| **Δ*gprH*Δ*gprI*** | *pyrG89*, Δ*gprH*::*pyro*^Af^, *pyroA4* , Δ*nkuA::argB (argB2),* Δ*gprI*::pyrG^Af^ | This work |
| **Δ*gprI*Δ*gprM*** | *pyrG89*, Δ*gprM*::*pyrG^Af^*, *pyroA4*, Δ*nkuA::argB (argB2),* Δ*gprI*::*pyro^Af^* | This work |
| ***ΔgprHΔgprIΔgprM*** | *pyrG89*, Δ*gprM*::*pyrG^Af^*, Δ*gprH*::*pyro^Af^, pyroA4*, Δ*nkuA::argB (argB2),* Δ*gprI*::*pyrG^Af^* | This work |
| ***VeA::GFP*** | *pyrG89, pyroA4,* Δ*nkuA::argB (argB2), veA::gfp::pyrG^Af^* | (17) |
| ***VeA::GFP mRFP::h2A*** | *pyrG89, pyroA4,* Δ*nkuA::argB (argB2), veA::gfp::pyrG^Af^ , mRFP::h2A::pRTA* | This work |
| ***ΔgprH VeA::GFP*** | *pyrG89, pyroA4,* Δ*gprH::pyro^Af^,* Δ*nkuA::argB (argB2), veA::gfp::pyrG^Af^* | This work |
| ***ΔgprH VeA::GFP mRFP::h2A*** | *pyrG89, pyroA4,* Δ*gprH::pyro^Af^,* Δ*nkuA::argB (argB2), veA::gfp::pyrG^Af^ , mRFP::h2A::prtA* | This work |
