# Supplementary material for: GPCR-mediated glucose sensing system regulates light-dependent fungal development and mycotoxin production
Source: PLoS Genet. 2019 Oct 14;15(10):e1008419. doi: 10.1371/journal.pgen.1008419 (PMC6812930; doi:10.1371/journal.pgen.1008419)
Supplement: S2 Table — (DOCX) [file pgen.1008419.s004.docx]

**Table S2.** List of primers used in this work.

| **Primer** | **Sequence (5' → 3')** |
| --- | --- |
| pRS426 gprH 5F | GGTTTTCCCAGTCACGACGGCTCAATTCGTAGCAAGAC |
| gprH pyro_5R | GACCCAACAACCATGATACC AGGCAACATGA CTGTTCG |
| gprH pyro 3F | CTGTCGATCATGTGGATGCTGATTGGGCCATGTAGAGTTATG |
| pRS426 gprH 3R | CTCTCTGGAAACAGCCGGAGATGATTCTGGTTCC |
| gprH tag pyrG rv | CGCATCAGTGCCTCCTCTCAGACAGAATCTAGTTCGTTAACCGGCGAGT |
| gprH 3UTR pyrG fw | GGTGAAGAGCATTGTTTGAGGCGAATTCGATTGGGCCATGTAGAGTT  ATG |
| An6680 pRS 5F | GTAACGCCAGGGTTTTCCCAGTCACGACGGACAGCTTCAATCACCATG |
| An6680 pyrG 5R | GTGCCTCCTCTCAGCAGATTGACGGTTCCTAGCAGCATCG |
| AN6680 PRS 3R | CTCTCTGGAAACAGCTAGCCACGTTCTACTGTAC |
| an6680 pyrG 3F | GAGCATTGTTTGAGGCGAATTCA |
| gprI_pRS 5F | GGTTTTCCCAGTCACGACGGAGATGACCACGCCGGCAACGCG |
| gprI pyro 5R | GACCCAACAACCATGATACCATATGAGCCTGAGGCGCTGCG |
| gprI pyro 3F | CTGTCGATCATGTGGATGCTCCGAGAGATCGGCATCATCTC |
| gprI pRS 3R | CTCTCTGGAAACAGCGCATTCCAGAGCCTCATGCGTAAACAAGC |
| gprI pyrG 5R | GCCTCCTCTCAGACAGAATTATGAGCCTGAGGCGCTGCGCAGGC |
| gprI pyrG 3F | GAGCATTGTTTGAGGCGAATTCCCGAGAGATCGGCATCAT |
| gprI tag pyrG rv | CGCATCAGTGCCTCCTCTCAGACAGAATCTACGTCTTCCTGTCAGCCT |
| gprI 3UTR pyrG fw | GGTGAAGAGCATTGTTTGAGGCGAATTCCCGAGAGATCGGCATCATC  TCAGC |
| pyrG_ Fw | ATTCTGTCTGAGAGGAGGCACTGATGCG |
| pyrG_ Rv | GAATTCGCCTCAAACAATGCTCTTCACC |
| pyro_ Fw | TGGTATCATGGTTGTTGGGTC |
| pyro_ Rv | AGCATCCACATGATCGACAG |
| prtA_F | GATCTGACAGACGGGCAATTG |
| prtA_R | CTATCATGGGGTGACGATGAGCCG |
| h2A 5UTR F_pRS426 | GTAACGCCAGGGTTTTCCCAGTCACGACGCAGCCAAGTTAGCTTCA  TAACC |
| 5UTR h2A + tag mRFP R | GATGACGTCCTCGGAGGAGGCCATGGTGATGTCCTGAGATGCGAAA  ACGACG |
| term. h2A_R | ATCTGGAGGGGACAGGCAGTTTAT |
| term. h2A + prtA_F | ATAAACTGCCTGTCCCCTCCAGATGATCTGACAGACGGGCAATTG |
| h2A 3UTR_F tag prtA | CGGCTCATCGTCACCCCATGATAGGGATAATGGCTTGATATGACC |
| h2A 3UTR_R tag pRS426 | GCGGTTAACAATTTCTCTCTGGAAACAGCCTTCTCAGTCGCTTCCTCTG |
| mRFP start F | GACATCACCATGGCCTCCTCCGAGGACGTCATC |
| h2A 1500UP_F | CGAGGAAGGGTATTTATACAACC |
| tubC_SYBR_Fw | AGCTGGCGGTAACAAATACG |
| tubC_SYBR_Rv | ACCTGATCCACCAATTCTGC |
| gprH_SYBR_Fw | GCAGCGAAGAACCTTTTGAC |
| gprH_SYBR_Rv | AGCAGGGCCACGAAAAATAG |
| gprM_SYBR fw | GGGCATTTATTTGTGGGTTG |
| gprM_SYBR rv | ACGCTATCACGGGTATCCAG |
| gprI_SYBR_new model_F | CGTTCTGCTACTCACCGAAC |
| gprI_SYBR_new model_R | CCTGGAAGCGACACAGTCC |
| aflR_SYBR_F | GCTCCAGATCCAAGGTCAAG |
| aflR_SYBR_R | CGTATTCGTCGGTGTTGTTG |
| veA_SYBR_F | GTCGGCAGTGGAATATGGAC |
| veA_SYBR_R | GAATCGGCGTAGAAGATGGA |
| stcU_SYBR_F | CGACAAGAAGATCACGGTCA |
| stcU_SYBR_R | CACTCATCCACCTGCTCATC |
| laeA_SYBR_F | CTATTCAGCCTCCGAACCAC |
| laeA_SYBR_R | GACACTACCGCAACCCATCT |
